# Ferroptosis: a novel pharmacological mechanism against multiple myeloma

**DOI:** 10.3389/fphar.2025.1606804

**Published:** 2025-07-15

**Authors:** Jingbo Shi, Yitong Lu, Wenjian Wei, Guodong Ma, Changnian Li, Lulu Li, Yaru Wang, Yan Wang, Ruirong Xu, Siyuan Cui

**Affiliations:** ^1^ Department of First Clinical Medical College, Shandong University of Traditional Chinese Medicine, Jinan, China; ^2^ Department of Hematology, Affiliated Hospital of Shandong University of Traditional Chinese Medicine, Jinan, China

**Keywords:** multiple myeloma, ferroptosis, pharmacological mechanism, natural products, antitumor effects

## Abstract

**Background:**

Multiple myeloma (MM) is a malignant disease characterized by the clonal proliferation of plasma cells in the bone marrow. Currently incurable, relapse and drug resistance remain significant challenges, necessitating the exploration of novel anti-MM agents. Ferroptosis, a form of cell death driven by iron-dependent lipid peroxidation, has emerged as a critical player in MM pathology and treatment. With advancing research, emerging evidence links ferroptosis to MM pathogenesis and therapeutic strategies. Natural products (NPs) and certain antitumor agents, owing to their broad bioactivities, demonstrate unique pharmacological advantages in MM therapy by targeting ferroptosis-related pathways.

**Purpose:**

This review systematically examines ferroptosis-related pathways in MM pathogenesis, focusing on pharmacological and toxicological mechanisms of natural products (NPs) and antitumor compounds in modulating ferroptosis-related pathways. It aims to provide novel insights and strategies for MM research and clinical therapy.

**Methods:**

We systematically retrieved data from PubMed, Web of Science, ScienceDirect, SciFinder, Scopus, and the China National Knowledge Infrastructure (CNKI) spanning database inception to March 2025, followed by taxonomic integrative analysis of NPs’ and antitumor compounds’ echanistic classifications.

**Results:**

NPs and antitumor compounds exert anti-MM effects via ferroptosis modulation, mechanistically mediated through: 1) lipid metabolism reprogramming; 2) ferritinophagy-driven iron homeostasis regulation; 3) Reactive oxygen species (ROS)-mediated oxidative stress potentiation; 4) autophagic activation; 5) Genes and proteins regulation.

**Conclusion:**

NPs and antitumor compounds demonstrate therapeutic potential against MM through multi-dimensional ferroptosis modulation, yet clinical translation faces two critical hurdles: 1) predominant focus on single-target mechanisms lacking systems pharmacology-level network analysis; 2) overreliance on *in vitro* models with insufficient clinical validation. Prioritize developing biomarkers and ferroptosis inducers to advance novel ferroptosis-targeting anticancer compounds.

## 1 Introduction

Multiple myeloma (MM), a hematologic malignancy characterized by clonal proliferation of bone marrow plasma cells, manifests clinically through CRAB criteria: hypercalcemia, renal dysfunction, anemia, and osteolytic lesions (van de Donk et al., 2021; Malard et al., 2024). Despite significant improvements in patient prognosis over recent decades through therapeutic advances—including proteasome inhibitors (PIs), immunomodulatory drugs (IMiDs), monoclonal antibodies, bispecific antibodies, and chimeric antigen receptor T-cell immunotherapy (CAR-T) (Rajkumar and Kumar, 2020), the disease remains incurable due to inevitable relapse and drug resistance during maintenance therapy. This underscores an urgent need for novel therapeutic targets and modalities.

Ferroptosis, an iron-dependent non-apoptotic cell death driven by excessive lipid peroxidation, was first characterized by Dixon et al., in 2012 through studies on erastin and RAS-selective lethal compounds (Dixon et al., 2012). Emerging as a pivotal research focus, ferroptosis has been implicated in diverse pathologies including hepatic disorders, breast cancer, cardiovascular diseases, and neurodegenerative conditions (Mahoney-Sánchez et al., 2021; Zhang et al., 2022; Cui et al., 2023; Yang F. et al., 2023), while demonstrating therapeutic potential in oncology (Liang et al., 2019; Lei et al., 2022). In MM pathogenesis, accumulating evidence links disease progression to dysregulated ferroptosis signaling pathways, suggesting opportunities for targeted drug development (Zhong et al., 2020; Li W. et al., 2022; Zhang et al., 2023). Furthermore, ferroptosis-related genes (FRGs) show promise as prognostic biomarkers for MM patients (Fu et al., 2022; Qin et al., 2022; Gao et al., 2023; Wang Q. et al., 2023).

**FIGURE 1:**
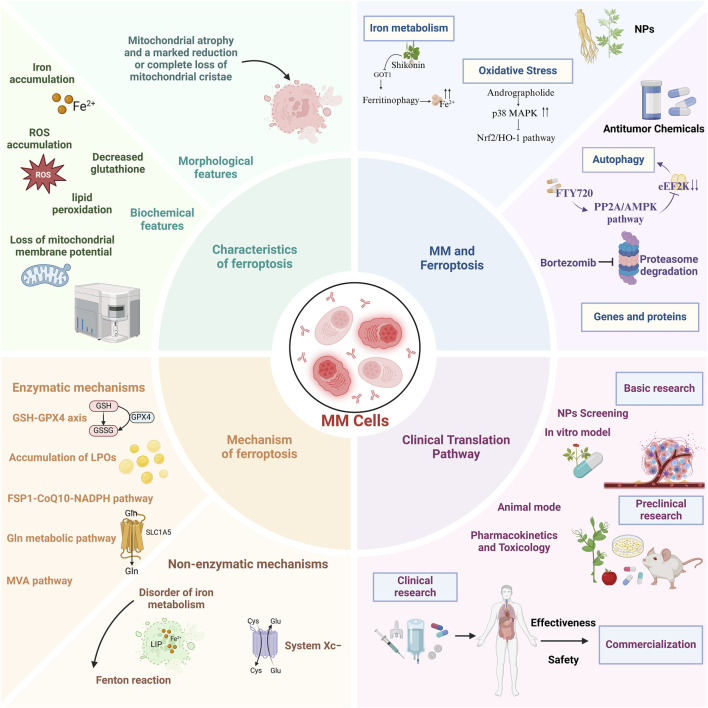
A comprehensive overview of the characteristics, mechanisms, role of ferroptosis in multiple myeloma, and potential clinical translational pathways.

This review systematically elucidates the molecular mechanisms of ferroptosis, its interplay with MM pathogenesis, and pharmacologic strategies leveraging ferroptosis modulation for MM intervention. Our synthesis aims to inform mechanistic investigations and advance clinical translation of ferroptosis-targeted therapies (Figure 1).

## 2 Review methodology

In accordance with the Preferred Reporting Items for Systematic Reviews and Meta - Analyses (PRISMA) guidelines, we conducted a systematic review to explore the regulatory role of ferroptosis in MM. Data were collected from research articles published up to March 2025, sourced from eight well - known databases: PubMed, Web of Science, ScienceDirect, Google Scholar, SciFinder, ResearchGate, Scopus, and the China National Knowledge Infrastructure (CNKI). The search terms used were “Multiple myeloma,” “Ferroptosis,” “Natural products,” “Molecular mechanisms,” and “Antitumor effects.” English - language publications, including scientific research, clinical trials, reviews, and meta - analyses, were considered for inclusion. Since this analysis did not involve human or animal subjects, ethical committee approval was not required. After excluding case reports and letters, and subsequent review of abstracts and full - texts, a total of 219 articles were included for evaluation in this manuscript.

## 3 Overview of ferroptosis

### 3.1 Evolutionary insights into ferroptosis

The conceptual foundation of ferroptosis was laid in 2003 when Dolma et al. identified erastin as a novel compound selectively lethal to RAS-mutated BJeLR fibroblasts through rapid, irreversible cytotoxicity (Dolma et al., 2003). Subsequent studies revealed the iron-dependent non-apoptotic death mechanism of RAS-mutated cancer cells induced by small molecules RSL3 and RSL5 (Yang and Stockwell, 2008). The term “ferroptosis” was formally proposed in 2012, with mechanistic studies demonstrating erastin’s dual action in HT-1080 fibrosarcoma models: mitochondrial voltage-dependent anion channel (VDAC) modulation and inhibition of the Xc system subunit solute carrier family 7 member 11 (SLC7A11), leading to glutathione depletion and iron-dependent lipid ROS accumulation (Dixon et al., 2012).

A pivotal 2014 study established glutathione peroxidase 4 (GPX4) as the central regulator of ferroptosis, with therapeutic implications validated in tumor xenografts (Yang et al., 2014). Concurrently, p53 was shown to enhance ferroptosis sensitivity by suppressing SLC7A11-mediated cysteine (Cys) uptake, while its acetylation-deficient mutant (p53 3 KR) retained regulatory capacity over ferroptosis under oxidative stress (Jiang et al., 2015). In 2017, acyl-CoA synthetase long-chain family member 4 (ACSL4) was identified as essential for ferroptosis execution by enriching ω6 polyunsaturated fatty acid (PUFA)-containing membranes (Doll et al., 2017). Subsequent work in 2019 uncovered an independent glutathione-bypassing ferroptosis suppression axis mediated by ferroptosis suppressor protein 1 (FSP1) via Coenzyme Q10 (CoQ10) (Doll et al., 2019).

Recent advances have uncovered novel regulatory networks in ferroptosis. The nuclear factor erythroid-2 related factor 2 (Nrf2)-HECT and RLD domain containing E3 ubiquitin protein ligase 2 (HERC2) axis has been shown to modulate cellular iron homeostasis and ferroptosis sensitivity through vesicle associated membrane protein 8 (VAMP8) regulation (Anandhan et al., 2023). Pharmacological targeting of emopamil binding protein (EBP) to block 7-dehydrocholesterol (7-DHC) biosynthesis demonstrates potent ferroptosis induction and antitumor efficacy, with pathway components including cytochrome p450 family 51 subfamily A member 1 (CYP51A1), EBP, and sterol C5-desaturase (SC5D) emerging as potential ferroptosis inhibitors (Freitas et al., 2024; Li Y. et al., 2024). These discoveries have propelled ferroptosis research from basic biological characterization to therapeutic exploration in diverse pathologies including oncology, neurodegenerative disorders, and ischemia-reperfusion injury, offering innovative perspectives for disease mechanism elucidation and intervention strategies (Chen X. et al., 2021; Ju et al., 2023; Wang Y. et al., 2023).

### 3.2 Morphological characteristics of ferroptotic cells

**FIGURE 2:**
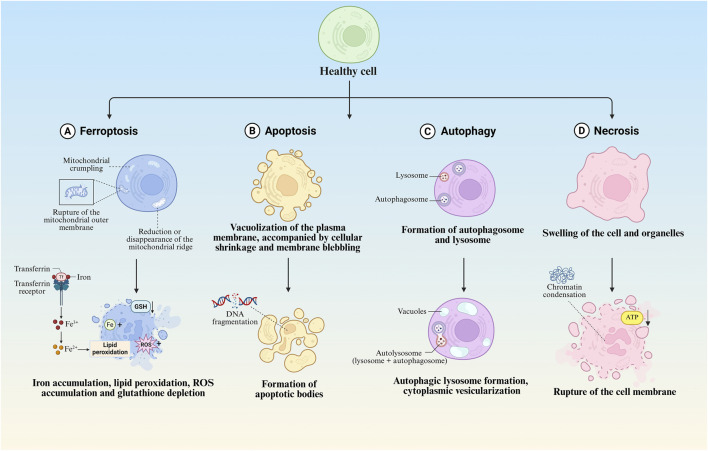
Morphological features of different regulated cell death modalities. **(A)** Morphological and biochemical characteristics of ferroptosis. **(B)** Morphological and biochemical characteristics of apoptosis. **(C)** Morphological and biochemical characteristics of autophagy. **(D)** Morphological and biochemical characteristics of necrosis.

Ferroptosis manifests distinct morphological alterations that differentiate it from other cell death modalities. Morphologically, ferroptotic cells exhibit cytoplasmic shrinkage, contrasting with apoptotic cell contraction through its association with intracellular lipid peroxide accumulation rather than membrane blebbing. Crucially, these cells preserve plasma membrane integrity and nuclear architecture, lacking characteristic apoptotic features such as chromatin margination, plasma membrane blebbing, or apoptotic body formation (Yagoda et al., 2007; Dixon et al., 2012; Yan H. et al., 2021), unlike autophagic cell death, they show neither double-membrane autophagic vesicles nor compromised membrane integrity (Liang et al., 2019). The pathognomonic ultrastructural features include mitochondrial atrophy with reduced cristae density and increased membrane condensation (Dixon et al., 2012; Stockwell et al., 2017) (Table 1; Figure 2).

**TABLE 1:** Hallmarks of ferroptosis and other regulated cell death (RCD) modalities: morphological, biochemical, and inducing factors.

RCD Types	Morphological features	Biochemical features	Inducing factors
Ferroptosis	Mitochondrial atrophy, accompanied by enhanced double-membrane density, disruption of the outer membrane, and a marked reduction or complete loss of mitochondrial cristaes (Yan et al., 2021b)	Iron accumulation, lipid peroxidation, ROS accumulation, glutathione depletion, increased NADPH oxidation, and loss of mitochondrial membrane potential (Li et al., 2020a; Liu et al., 2022b)	Disruptions in iron metabolism, the accumulation of lipid peroxides, and associated factors
Apoptosis	Vacuolization of the plasma membrane, accompanied by nuclear rupture and folding, chromatin condensation, cellular shrinkage, the formation of apoptotic bodies, and pronounced chromatin compaction (VanHook, 2023)	Caspase activation and DNA fragmentation (Chen et al., 2018; Li et al., 2020a)	Regulation of relevant genes under non-pathological conditions
Autophagy	The appearance of autophagic vacuoles, which progressively enclose to form autophagosomes and ultimately develop into autolysosomes (Zhao et al., 2024)	LC3-I is converted into LC3-II, and autophagic substrates are degraded (Chen et al., 2018; Li et al., 2020a)	Organelle damage, nutrient deficiency, metabolic disorders, microbial infections, and others
Necrosis	The nucleus undergoes condensation, fragmentation, and dissolution; the cell membrane ruptures, accompanied by swelling of the cytoplasm and organelles, as well as chromatin condensation (Kuroe et al., 2021)	ATP levels decline, accompanied by the activation of RIP1, RIP3, and MLKL (Chen et al., 2018; Li et al., 2020a)	Severe pathological damage

NADPH, Nicotinamide adenine dinucleotide phosphate; LC3-I, Microtubule-associated proteins light chain 3-I; LC3-II, Microtubule-associated proteins light chain 3-II; RIP1, Receptor-interacting protein 1; RIP3, Receptor-interacting protein 3; MLKL, Mixed-lineage kinase domain-like.

## 4 Mechanism of ferroptosis

As an emerging cell death modality, ferroptosis has evolved from the convergence of research in amino acid/lipid metabolism, iron homeostasis, redox biology, selenium regulation, and programmed cell death. Its execution involves a dual-pathway mechanism: enzymatic peroxidation mediated by arachidonate lipoxygenases (ALOXs) and non-enzymatic Fenton reactions. These processes generate cytotoxic phospholipid hydroperoxides that induce membrane damage, ultimately driving ferroptotic cell death.

### 4.1 Enzymatic mechanisms

#### 4.1.1 The glutathione (GSH)-GPX4 axis

Ferroptosis is characterized by the hallmark feature of intracellular lipid peroxide accumulation, with the glutathione-glutathione peroxidase 4 (GSH-GPX4) axis playing a pivotal role in suppressing lipid peroxidation. GPX4 represents the sole enzymatic system capable of reducing lipid hydroperoxides in mammalian cells, requiring reduced GSH as an essential cofactor for neutralizing lipid peroxides (LPOs). Depletion of GSH or inactivation of GPX4 disrupts this equilibrium, leading to lipid peroxide accumulation and subsequent ferroptosis induction.

Under physiological conditions, cellular homeostasis is maintained through a delicate balance between pro-oxidative systems (comprising iron ions and reactive oxygen species) and antioxidant defenses mediated by the System Xc-/GSH/GPX4 axis. This equilibrium becomes disrupted under pathological states, triggering ferroptotic cell death. As the fourth selenoprotein in the glutathione peroxidase family, GPX4 (originally termed PHGPX for its phospholipid hydroperoxide-reducing capacity) serves as the unique enzymatic regulator of lipid peroxidation through its distinctive structural and functional properties (Xie et al., 2023). The enzyme exerts its protective effects by catalyzing the conversion of phospholipid hydroperoxides (PLOOH) into non-reactive fatty alcohols (PLOH), thereby preventing oxidative membrane damage (Nishida Xavier da Silva et al., 2022), this selenium-dependent peroxidase specifically utilizes reduced GSH to maintain membrane integrity through two critical reactions: reduction of lipid hydroperoxides to corresponding alcohols, and detoxification of hydrogen peroxide to water (Ma et al., 2022; Zhang W. et al., 2024). The catalytic cycle of GPX4 involves dynamic selenocysteine redox transitions. The active site selenol becomes oxidized to selenenic acid during peroxide reduction, subsequently regenerated through GSH-mediated reduction that produces oxidative glutathione (GSSG). This mechanism establishes GSH as an indispensable factor for sustaining GPX4 enzymatic activity (Forcina and Dixon, 2019; Yan H. et al., 2021; Tuncer and Hacioglu, 2024).

#### 4.1.2 Accumulation of LPOs

Ferroptosis represents a distinct form of cell death mechanistically linked to dysregulated lipid metabolism, where the pathological accumulation of LPOs serves as a critical determinant. Lipoxygenases (LOXs), non-heme iron-containing enzymes, catalyze the stereospecific oxygenation of free polyunsaturated fatty acids (PUFAs) into lipid hydroperoxides through molecular oxygen incorporation. This enzymatic activity plays a pivotal role in driving ferroptosis progression (Liu et al., 2023; Mortensen et al., 2023). Membrane-incorporated PUFAs serve as primary targets for ROS-mediated oxidation, with their oxidative susceptibility increasing proportionally to the number of conjugated double bonds (Rouzer and Marnett, 2003), following the initiation of oxidation, free radicals exhibit dual propagation mechanisms: intramolecular migration within lipid bilayers or intermolecular chain reactions amplifying oxidative damage (Samovich et al., 2024). Notably, arachidonic acid (AA) and adrenal acid (AdA), the most oxidation-prone PUFA species, demonstrate particular vulnerability to this peroxidation cascade.

Emerging evidence identifies AA/AdA-derived phosphatidylethanolamine (PE) as a pivotal substrate for ferroptotic lipid peroxidation (Kagan et al., 2017; Samovich et al., 2024). The enzymatic cascade begins with ACSL4 converting free AA/AdA into AA-CoA/AdA-CoA, followed by lysophosphatidylcholine acyltransferase 3 (LPCAT3)-mediated esterification to generate AA-PE/AdA-PE. These phospholipid conjugates undergo iron/LOX-dependent oxidation to PLOOH, thereby initiating membrane peroxidation and ferroptotic execution (Yang et al., 2016; Dierge et al., 2021). Contrastingly, monounsaturated fatty acids (MUFAs) are preferentially incorporated into membrane phospholipids via ACSL3-mediated acyl-CoA synthesis. Exogenous MUFAs suppress ferroptosis through ACSL3 activation, effectively attenuating lipid ROS accumulation (Ru et al., 2024). Notably, Huang et al. identified AS-252424 as a competitive inhibitor targeting the Gln464 residue of ACSL4, effectively blocking enzymatic activity and ferroptotic progression (Huang et al., 2024). Furthermore, redox enzymes contribute to lipid peroxidation by transferring electrons from NADPH to oxygen, generating hydrogen peroxide. This process facilitates Fenton reactions with labile iron pools, thereby amplifying oxidative chain reactions that drive ferroptosis (Yan B. et al., 2021).

#### 4.1.3 The FSP1-CoQ10-NADPH pathway

Researchers identified genes in tumor cells capable of compensating for the deficiency of GPX4, a key enzyme in ferroptosis, through expression cloning. Apoptosis - inducing factor mitochondria - associated 2 (AIFM2), a lipid - binding protein, was recognized as a novel anti - ferroptosis gene, Bersuker et al. renamed AIFM2 as FSP1 (Bersuker et al., 2019; Doll et al., 2019). The inhibition of ferroptosis by FSP1 is mediated by CoQ10. Specifically, the reduced form of CoQ10 can scavenge lipid peroxyl radicals, which mediate lipid peroxidation. FSP1 can regenerate CoQ10 by catalyzing NADPH (Doll et al., 2019). In detail, in the FSP1 - CoQ10 - NADPH pathway, FSP1, as an effective ferroptosis inhibitor, reduces CoQ10 on the plasma membrane to ubiquinol. This process either directly inhibits lipid peroxidation or indirectly promotes the regeneration of tocopherol radicals (vitamin E), a natural chain - breaking antioxidant.

Recent studies have shown that FSP1 possesses a unique carboxyl - terminal domain. This domain can mediate the functional dimerization of FSP1 and the formation of two active sites, which are crucial for the catalytic activity and ferroptosis - inhibitory property of FSP1. *In vitro*, in the presence of oxygen and NADPH, FSP1 can catalyze the generation of hydrogen peroxide and convert FAD into 6 - hydroxy - FAD. 6 - hydroxy - FAD is not only an active co - factor of FSP1 but also an effective radical - trapping antioxidant that can directly inhibit ferroptosis in cells (Lv et al., 2023). As a glutathione - independent ferroptosis suppressor, FSP1, by regulating the CoQ10 and NADPH pathways, collaborates with GPX4 to inhibit phospholipid peroxidation and ferroptosis, providing new targets and strategies for cancer treatment.

#### 4.1.4 Glutamine metabolic pathway

The glutamine (Gln) metabolic pathway plays a critical role in regulating ferroptosis. Gln is primarily taken up by cells through solute carrier family 1 member 5 (SLC1A5) and subsequently catabolized to glutamate (Glu) by glutaminase (GLS) (Bodineau et al., 2022; Li S. et al., 2023). Glu is then converted to α-ketoglutarate (α-KG) via glutamate dehydrogenase (GLUD1), which enters the tricarboxylic acid (TCA) cycle (Yao et al., 2021), α-KG accumulation promotes oxidative stress by enhancing ROS production and lipid peroxidation, thereby inducing ferroptosis (Gao et al., 2015). Notably, Glu triggers mitochondrial structural and functional alterations, including membrane potential depolarization and reduced electron transport chain activity, further contributing to lipid ROS accumulation. Furthermore, Glu serves as a precursor for glutathione (GSH) synthesis. As the primary intracellular antioxidant, GSH scavenges excessive ROS to mitigate lipid peroxidation, a mechanism crucial for ferroptosis prevention (Kanaan et al., 2024).

Gln participates in the TCA cycle and energy metabolism, providing biosynthetic energy substrates. Under oxidative stress, metabolic reprogramming may influence cellular survival and ferroptosis susceptibility (Suzuki et al., 2022), Gln also modulates iron homeostasis by regulating its uptake and storage, thereby indirectly controlling iron overload risks. Excessive iron accumulation potentiates ROS generation, ultimately triggering ferroptosis (Chen et al., 2024). A recent study demonstrated that Gln protects nucleus pulposus (NP) cells from tert-butyl hydroperoxide (TBHP)-induced ferroptosis and extracellular matrix (ECM) degradation. This protective effect occurs through enhanced Nrf2 stabilization via inhibition of ubiquitin-proteasome degradation, coupled with suppressed lipid oxidation (Fu et al., 2024; Wu J. et al., 2024).

#### 4.1.5 Mevalonate pathway

The mevalonate (MVA) pathway plays a pivotal role in ferroptosis regulation by modulating selenocysteine-tRNA maturation, thereby influencing GPX4 biosynthesis. Selenocysteine (Sec), an essential catalytic residue in GPX4’s active site, is indispensable for its antioxidant function (Chen et al., 2020). The MVA pathway initiates from acetyl-CoA and sequentially generates isopentenyl pyrophosphate (IPP) through reductase-mediated steps, with critical enzymatic involvement from mevalonate kinase (MVK). Crucially, MVA-derived metabolites including IPP and CoQ10 regulate Sec-tRNA maturation and subsequent GPX4 synthesis. The incorporation of Sec during GPX4 translation proves vital for maintaining its redox-protective capacity (Wu et al., 2022). Pharmacological inhibition of the MVA pathway impairs Sec-tRNA biogenesis, leading to GPX4 inactivation, diminished cellular antioxidant defenses, lipid peroxide accumulation, and ultimately ferroptosis induction (Chen et al., 2020).

Recent studies demonstrate that the bisphosphonate alendronate synergistically enhances paclitaxel efficacy, suppresses tumor metastasis, and modifies cytotoxic mechanisms in cancer therapy. Mechanistically, alendronate inhibits mevalonate metabolism, induces mitochondrial morphological alterations, disrupts redox homeostasis, and promotes accumulation of mitochondrial ROS and LPOs - ultimately triggering tumor cell ferroptosis. Notably, hepatocellular carcinoma (HCC) cells overexpressing miR-612 exhibit heightened ferroptosis sensitivity, with miR-612 potentiating lipid ROS accumulation (Song G. et al., 2024). Xing et al. further revealed that miR-612 promotes HCC ferroptosis through HADHA-mediated regulation of the mevalonate pathway, downregulating CoQ10 while elevating intracellular PUFA levels and lipid peroxidation, thereby inhibiting HCC proliferation and metastasis (Xing et al., 2023). Complementary findings by Tang et al. demonstrate that pitavastatin induces ferroptosis in malondialdehyde (MDA)-MB-231 triple-negative breast cancer cells via mevalonate pathway inhibition, with exogenous mevalonate supplementation partially reversing pitavastatin-induced cytotoxicity and the reduced expression of GPx4/FSP1 (Tang W.-J. et al., 2024).

### 4.2 Non-enzymatic mechanisms

#### 4.2.1 Disorder of iron metabolism

Iron metabolism dysregulation is intrinsically linked to ferroptosis pathogenesis. Under physiological conditions, systemic iron homeostasis is dynamically regulated through coordinated expression of transferrin (Tf), transferrin receptor 1 (TfR1), ferroportin 1 (FPN1), and hepatic iron regulatory proteins (Galy et al., 2024). Tf, a single-chain polypeptide serving as the principal iron-transport protein, serves as a biochemical indicator of systemic iron metabolism (Zhang D. et al., 2024). TfR1, a transmembrane protein mediating cellular iron uptake through Tf-bound iron internalization, has been identified as a specific ferroptosis biomarker (Feng et al., 2020). The iron transport cascade initiates with extracellular Fe^3+^ binding to Tf, followed by TfR1-mediated endocytosis. Within endosomal vesicles, Fe^3+^ undergoes six-transmembrane epithelial antigen of prostate 3 (STEAP3)-catalyzed reduction to Fe^2+^, subsequently exported to cytosol via divalent metal transporter 1 (DMT1) (Wang H. et al., 2023). When iron-binding capacity approaches saturation, excess Fe^2+^ complexes are sequestered in the labile iron pool (LIP) through ferritin binding. LIP-derived Fe^2+^ participates in Fenton reactions, generating hydroxyl radical species that drive membrane lipid peroxidation and subsequent ferroptosis (Morales and Xue, 2021), furthermore, ferritin-bound iron undergoes Nuclear receptor coactivator 4 (NCOA4)-mediated autophagic degradation in lysosomes, releasing free Fe^2+^ into the intracellular compartment (Chen et al., 2022b).

The Fenton reaction, mediated by Fe^2+^ and hydrogen peroxide, generates hydroxyl radicals that initiate non-enzymatic lipid peroxidation by abstracting hydrogen atoms from lipids, forming lipid radicals (Sun et al., 2022). Notably, extracellular conversion of FPN1-exported Fe^2+^ into less redox-active Fe^3+^ is facilitated by ceruloplasmin (CP). CP deficiency disrupts this process, leading to pathological Fe^2+^ accumulation and subsequent ferroptosis induction (Shang et al., 2020).

#### 4.2.2 System Xc−

System Xc^−^, a critical intracellular antioxidant component, is an amino acid antiporter composed of the light chain SLC7A11 (xCT) and heavy chain SLC3A2 (4F2hc), which are interconnected by disulfide bonds (Li F.-J. et al., 2022; Aboushousha et al., n.d.). This transporter operates via the Glu and Cys concentration gradient across cellular membranes, primarily facilitating extracellular Cys import and intracellular Glu export in a 1:1 stoichiometric exchange. This mechanism is indispensable for GSH synthesis (Wu et al., 2021; Li F.-J. et al., 2022). Within this pathway, ingested Cys is catalyzed by thioredoxin reductase 1 (TrxR1) to convert it to Cys, followed by conjugation with Glu via glutamate-cysteine ligase (GCL) to yield γ-glutamyl-L-cysteine. This intermediate subsequently combines with glycine to complete GSH biosynthesis, thereby inhibiting ferroptosis (Li F.-J. et al., 2022; Liu J. et al., 2022; Saini et al., 2023; Zhang G. et al., 2024).

System Xc^−^ serves as the pivotal upstream regulator of the System Xc^−^/GSH/GPX4 axis, with its activity primarily governed by SLC7A11 expression. SLC7A11 downregulation diminishes System Xc^−^ activity, promoting oxidative stress-mediated ferroptosis. Conversely, SLC7A11 upregulation enhances cellular resistance to ferroptosis, a mechanism implicated in tumor chemoresistance (Wang et al., 2020; Xue et al., 2024).

#### 4.2.3 Fenton reaction

The Fenton reaction, a redox interaction between iron and hydrogen peroxide, generates hydroxyl radicals—highly reactive and cytotoxic species that drive ferroptosis by abstracting hydrogen atoms from lipids to initiate non-enzymatic lipid peroxidation (He et al., 2020; Abe et al., 2022). Henning et al. demonstrated that sodium iodate (SI) and dimethyloxalylglycine (DMOG) treatment reduced superoxide dismutase activity in retinal pigment epithelial (RPE) cells, revealing hypoxia-enhanced Fenton reactions as a mechanism exacerbating ferroptosis in RPE cells (Henning et al., 2022). Recent studies indicate phenolic compounds induce ferroptosis in multiple cell types by forming iron complexes that potentiate Fenton reactivity, leading to hydroxyl radical overaccumulation and ferroptosis-like death (Sui et al., 2024). Furthermore, Fenton reaction-based nanocatalytic therapies show promise for targeting ferroptosis-related pathways in cancer treatment (Xing et al., 2021; Zhu et al., 2022; Zhao et al., 2023; Song Q. et al., 2024).

**FIGURE 3:**
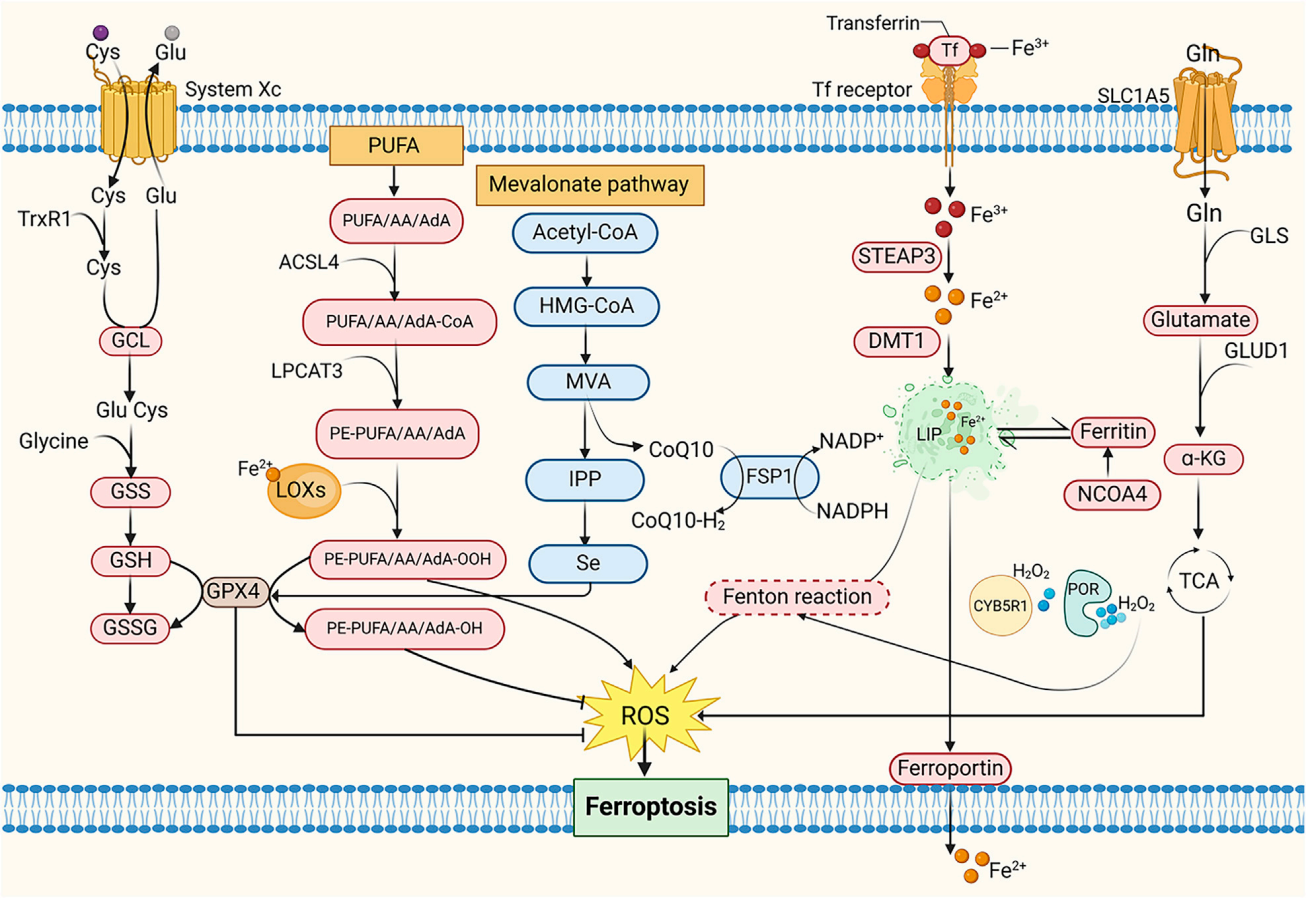
The mechanism of ferroptosis. Cys, Cysteine; Glu, Glutamate; TrxR1, Thioredoxin reductase 1; GCL, Glutamate-cysteine ligase; GSS, Glutathione synthetase; GSH, Glutathione; GSSG, Oxidative glutathione; PUFA, Polyunsaturated fatty acid; AA, Arachidonic acid; AdA, Adrenal acid; ACSL4, Acyl-CoA synthetase long-chain family member 4; LPCAT3, Lysophosphatidylcholine acyltransferase 3; PE, Phosphatidylethanolamine; LOXs, Lipoxygenases; MVA, Mevalonate; IPP, Isopentenyl pyrophosphate; HMG-CoA, 3-Hydroxy-3-Methyl-Glutaryl-CoA; CoQ10, Coenzyme Q10; FSP1, Ferroptosis suppressor protein 1; NADPH, Nicotinamide adenine dinucleotide phosphate; Tf, Transferrin; STEAP3, Six-transmembrane epithelial antigen of prostate 3; DMT1, Divalent metal transporter 1; LIP, Labile iron pool; NCOA4, Nuclear receptor coactivator 4; CYB5R1, NADH-cytochrome b5 reductase; POR, Cytochrome P450 reductase; Gln, Glutamine; SLC1A5, Solute carrier family 1 member 5; GLS, Glutaminase; GLUD1, Glutamate dehydrogenase; α-KG, α-ketoglutarate; TCA cycle, Tricarboxylic acid cycle.

Given that tumor cells exhibit elevated ROS levels due to accelerated metabolic and proliferative activity, ferroptosis—a process intimately linked to redox imbalance—may offer a targeted therapeutic approach for MM and other cancers (Chiang et al., 2018; Du et al., 2023). Rajkumar et al. demonstrated the anti-proliferative effects of ferrotoxic interventions in MM murine models (Rajkumar et al., 2014). Subsequent studies revealed heightened sensitivity of MM cell lines to ferroptosis, with combined bortezomib and iron chelation exhibiting superior efficacy in suppressing disease progression compared to monotherapy, these findings collectively underscore the critical role of ferroptosis in MM pathogenesis (Campanella et al., 2013; Bordini et al., 2017) (Figure 3).

## 5 Mechanistic differences of ferroptosis in normal and cancerous tissues

Ferroptosis, an iron-dependent lipid peroxidation-driven form of cell death, exhibits significant differences in its mechanisms between normal and cancerous tissues (Jiang X. et al., 2021). These differences are closely related to cell type characteristics, redox status, and the local microenvironment, which directly determine the contrasting roles of inducers and inhibitors in disease therapy.

In normal tissues, ferroptosis is typically associated with cellular stress responses, antioxidant defense mechanisms, and immune regulation. For example, in neurodegenerative diseases such as Alzheimer’s and Parkinson’s diseases, excessive oxidative damage in neuronal cells may accelerate disease progression by inducing ferroptosis, making ferroptosis inhibitors potential therapeutic agents. Ferroptosis inhibitors can slow down neuronal cell death and protect nerve function (Yang et al., 2022; Ryan et al., 2023; Zhang T.-C. et al., 2024).

In contrast, cancerous tissues exhibit a distinct scenario. Tumor cells often possess a strong antioxidant capacity to counteract redox imbalance. Due to the generally higher metabolic activity in cancer cells and the increased levels of ROS associated with cancer progression, they may be more susceptible to ferroptosis (Lei et al., 2022). Furthermore, studies have shown that tumor cells typically require higher iron availability, which may further increase their sensitivity to ferroptosis (Torti and Torti, 2013; Manz et al., 2016). Ferroptosis inducers can promote the accumulation of intracellular iron and lipid peroxides, activating the ferroptosis pathway and leading to tumor cell death. Therefore, ferroptosis inducers are considered a promising therapeutic strategy, especially for treating refractory cancers. By modulating ferroptosis induction or inhibition, different therapeutic effects can be achieved in various disease microenvironments, further highlighting the complexity of ferroptosis in different tissue types.

## 6 MM and ferroptosis

### 6.1 Lipid metabolism pathway

#### 6.1.1 AA pathway

Studies have demonstrated significant reductions in key PUFAs, including ferroptosis-inducing AA, within bone marrow aspirates of MM patients (Chen et al., 2023). Elevated expression of ferroptosis-suppressing genes correlates with reduced progression-free survival (PFS) and overall survival (OS) in MM patients (Su et al., 2022). Co-culture experiments of murine MM cells with bone marrow adipocytes revealed that MM cells upregulate fatty acid transport proteins 1 and 4 (FATP1, FATP4) to acquire PUFAs from neighboring adipocytes. PUFA exposure exerts dose-dependent effects on MM cells, with high concentrations triggering lipotoxicity via ferroptosis activation (Panaroni et al., 2022). These findings collectively implicate ferroptosis-mediated lipid metabolism dysregulation in MM pathogenesis. Free fatty acids (FFAs) exhibit dual regulatory roles: low concentrations promote MM proliferation, whereas elevated levels induce ferroptosis-dependent cytotoxicity. Notably, bone marrow lipid content is markedly increased in precursor MM states (MGUS and SMM) compared to healthy controls (Panaroni et al., 2022). AA administration dose-dependently suppresses proliferation and viability in human MM cell lines (MM1S, H929, U266) and enhances apoptosis rates in SCID mouse xenograft models. Therapeutic targeting of ferroptosis may yield superior efficacy during early disease stages (MGUS and SMM) (Panaroni et al., 2018). Paradoxically, while high AA concentrations induce MM cell death, subtoxic levels stimulate tumor growth through FATP-mediated FA uptake and adipocyte lipolysis (Panaroni et al., 2022).

Intriguingly, MM plasma cells exhibit elevated GPX4 and xCT levels compared to normal counterparts. Given their critical roles in ferroptosis suppression, these antioxidant regulators represent promising therapeutic targets for counteracting ROS-mediated cytotoxicity (Mynott et al., 2023). Precise determination of AA’s physiological thresholds is essential to maintain anti-tumor efficacy without triggering pro-survival adaptations.

#### 6.1.2 ACSL4 pathway

ACSL4 is aberrantly overexpressed in MM cell lines and patient-derived samples compared to healthy donors. This dysregulation exerts dual biological effects: ACSL4 enhances fatty acid accumulation by modulating lipid metabolism regulators (e.g., c-Myc and sterol regulatory element-binding proteins, SREBPs) to drive MM proliferation (Liang et al., 2023). However, as a critical ferroptosis driver, ACSL4 governs MM cell sensitivity to ferroptosis inducers like RSL3, with elevated ACSL4 levels potentiating ferroptotic vulnerability (Zhang et al., 2023). ACSL4 knockdown suppresses MM proliferation and attenuates intracellular fatty acid accumulation, potentially via modulation of lipid metabolic regulators. Crucially, ACSL4 silencing confers resistance to ferroptosis, directly linking its expression levels to cellular ferroptotic sensitivity (Zhang et al., 2023). These mechanistic insights position ferroptosis induction as a promising therapeutic strategy for MM, where pharmacological modulation of ACSL4 activity/expression could augment therapeutic efficacy.

### 6.2 Iron metabolism pathway

Iron overload has been shown to impair MM cell proliferation, an effect reversible by the ferroptosis inhibitor ferrostatin-1. High-dose ferric ammonium citrate (FeAC) triggers MM cell death with concurrent elevation of MDA - a lipid peroxidation byproduct and hallmark of ferroptosis, establishing iron metabolism as a critical modulator in MM pathophysiology (Bordini et al., 2020; Camiolo et al., 2020). Mechanistically, free iron accumulation induces dose-dependent aggregation of polyubiquitinated proteins in iron-sensitive MM H929 cells, while suppressing chymotrypsin-like proteasomal activity in cell lysates. This dual mechanism suggests that iron overload may potentiate proteasome inhibitor efficacy through lipid oxidation and proteostasis disruption, providing a rationale for combinatorial therapeutic strategies (Bordini et al., 2020; Camiolo et al., 2020). Iron homeostasis dysregulation in MM is further linked to altered hepcidin expression patterns. Although hepcidin (a hepatic iron-regulatory peptide) typically restricts systemic iron release (Nemeth and Ganz, 2023), MM patients exhibit paradoxical hepcidin downregulation or functional impairment during iron overload, creating a self-reinforcing cycle of iron accumulation that exacerbates disease progression (Maes et al., 2010; Banaszkiewicz et al., 2022).

### 6.3 Oxidative stress pathway

Emerging evidence demonstrates that andrographolide, a natural bioactive compound, induces ferroptosis in MM cells via P38-mediated suppression of the Nrf2/heme oxygenase-1 (HO-1) signaling axis, thereby triggering oxidative stress (Li W. et al., 2023). Mechanistically, Nrf2 serves as the master transcriptional regulator of antioxidant responses, orchestrating cellular iron homeostasis, redox balance, and mitochondrial function through transcriptional regulation of FPN1, GPX4, and HO-1. Crucially, Nrf2 knockout models exhibit diminished SLC7A11 and HO-1 protein expression, which potentiates ferroptotic susceptibility (Dong et al., 2020).

### 6.4 Autophagy pathway

Beclin-1, a core autophagy-related protein, drives autophagosome formation, while UNC-51 like kinase 1 (ULK1) coordinates autophagic initiation through its kinase complex (Kim et al., 2011). Notably, protein phosphatase 2A (PP2A) and AMP-activated protein kinase (AMPK) modulate the phosphorylation status of Beclin-1 to regulate autophagic flux (Peris et al., 2023). PP2A specifically dephosphorylates Beclin-1 at Ser90, whereas AMPK–activated under energy stress–phosphorylates ULK1 to initiate autophagy, and targets Beclin-1 at Ser91/Ser94 residues (Kim et al., 2013; Park et al., 2023). Functionally, the PP2A-AMPK axis orchestrates autophagic-ferroptotic crosstalk in MM cells. PP2A activation via dephosphorylation of its catalytic subunit C at Tyr307 induces AMPK inactivation through Thr172 dephosphorylation. This cascade attenuates eukaryotic elongation factor 2 kinase (eEF2k) activity, thereby relieving its inhibition of eEF2 to enhance energy expenditure and drive dual autophagic-ferroptotic cell death (Zhong et al., 2020). CRISPR/Cas9-mediated knockout of autophagy regulators ATG5/ATG7 in RPMI-8226 MM cells significantly increased cell viability compared to wild-type controls upon RSL3-induced ferroptosis, confirming autophagy’s synergistic role in ferroptotic execution (Li and Lyu, 2024).

### 6.5 Other regulatory mechanisms

The interplay between ferroptosis and epigenetic regulation has garnered increasing attention due to their shared associations with oxidative stress and dysregulated iron metabolism. Induction of ferroptosis in MM cells upregulates key genes implicated in cellular stress, death, inflammation, and fatty acid metabolism, including ferritin heavy chain 1 (FTH1), HO-1, and SLC7A11. Studies have demonstrated that ferroptosis induction in MM cells triggers DNA methylation and histone modification changes associated with cellular senescence. Logie et al. revealed that MM1 cells exhibit sensitivity to both the ferroptosis inducer RSL3 and epigenetic reprogramming. Liquid chromatography-tandem mass spectrometry (LC-MS/MS) analysis identified the formation of non-heme iron-histone complexes and altered histone modifications linked to DNA repair and senescence. Using EPIC BeadChip profiling, the authors observed significant DNA methylation alterations in ferroptotic MM cells, enriched in genes regulating cell cycle progression and senescence, such as nuclear receptor subfamily 4 group A member 2 (NR4A2). These findings highlight the connection between ferroptosis and epigenomic stress responses, underscoring the therapeutic potential of ferroptosis-inducing agents (Logie et al., 2021).

Emerging evidence indicates that the crosstalk between bone marrow mesenchymal stromal cells (BMSCs) and MM cells enhances ferroptosis susceptibility in the latter. BMSCs elevate intracellular iron levels in MM cells, thereby activating steroid biosynthesis pathways such as lanosterol production. Lanosterol, a major source of ROS in MM cells, is regulated by the CD40 ligand (CD40L)-CD40 receptor interaction, which serves as a critical signaling axis. BMSCs modulate the CD40/CD40L pathway to influence iron homeostasis and lanosterol synthesis in MM cells, driving ROS accumulation and ferroptosis (Jiang et al., 2024). A recent study further revealed that leukocyte immunoglobulin-like receptor B1 (LILRB1), through complex formation with other proteins, promotes low-density lipoprotein and cholesterol uptake. LILRB1 deficiency disrupts cholesterol uptake but activates compensatory cholesterol biosynthesis to maintain cellular cholesterol homeostasis. This shift reduces levels of the anti-ferroptotic metabolite squalene, rendering MM cells more prone to ferroptosis. Given its role in safeguarding MM cells against ferroptosis via cholesterol regulation, LILRB1 represents a promising therapeutic target (Xian et al., 2024).

**FIGURE 4:**
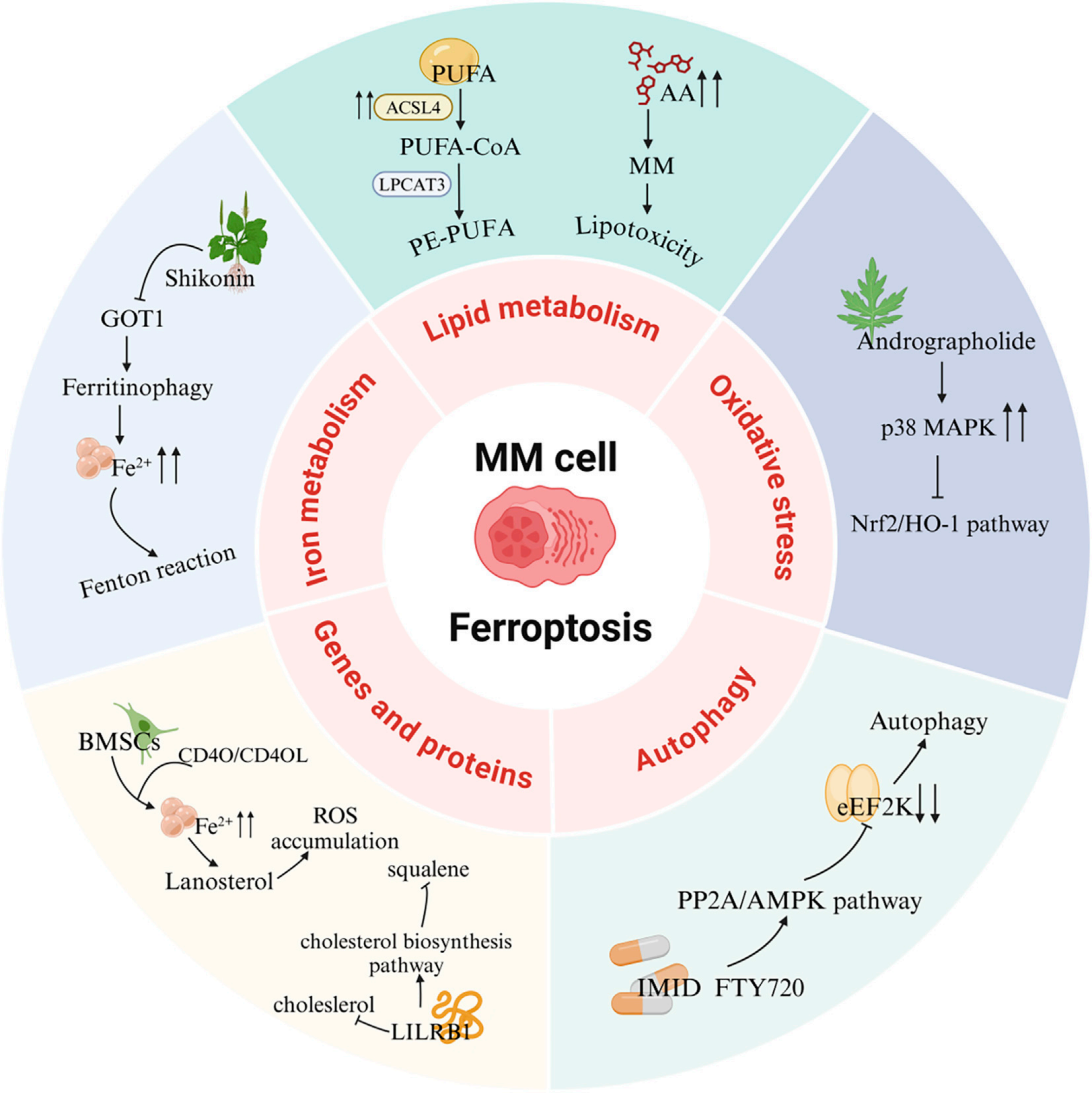
Ferroptosis signaling pathways in MM. PUFA, Polyunsaturated fatty acid; ACSL4, Acyl-CoA synthetase long-chain family member 4; LPCAT3, Lysophosphatidylcholine acyltransferase 3; AA, Arachidonic acid; MAPK, Mitogen-activated protein kinase; Nrf2, Nuclear factor E2-related factor 2; HO-1, Heme oxygenase-1; eEF2K, eukaryotic elongation factor 2 kinase; PP2A, Protein phosphatase 2A; AMPK, AMP-activated protein kinase; IMID, Immunomodulatory drug; FTY720, Fingolimod; BMSCs, Bone marrow mesenchymal stromal cells; LILRB1, Leukocyte immunoglobulin-like receptor B1; GOT1, Glutamic-oxaloacetic transaminase 1.

A recent study revealed that mitochondrial metabolic kinase PCK2 phosphorylates and activates ACSL4, driving phospholipid remodeling linked to ferroptosis. Tumor-reprogrammed cells (TRCs), characterized by stem-like properties, downregulate PCK2 expression to adopt a ferroptosis-resistant state, suggesting that TRCs evade radio- and chemotherapy by reducing ferroptosis susceptibility. These findings highlight novel insights into ferroptosis in malignant tumors (Li Z. et al., 2024). Fu et al. investigated the clinical and biological relevance of ferroptosis-related genes in MM. Their prognostic model underscored the critical role of ferroptosis in MM patient outcomes. *In vitro* experiments demonstrated synergistic antitumor efficacy of erastin and doxorubicin, mediated by GPX4 degradation and excessive ROS production, which collectively reduced viability of NCI-H929 and RPMI-8226 cells (Fu et al., 2022). Furthermore, glutamine blockade combined with radiotherapy (RT) induces immunogenic ferroptosis in tumor cells. Mechanistically, RT activates the interferon signaling pathway to upregulate interferon regulatory factor 1 (IRF1), an effect amplified by glutamine inhibition. IRF1 drives transferrin receptor expression, elevates intracellular Fe^2+^ levels, and disrupts iron homeostasis, thereby promoting ferroptosis. The therapeutic potential of this axis in hematologic malignancies warrants further exploration (Song A. et al., 2024) (Figure 4).

## 7 Ferroptosis-based treatment strategies for MM

### 7.1 Active ingredients of natural products

The partial inhibition of cell death by Ferrostatin-1, Liproxstatin-1, or Deferoxamine, alongside evidence that natural product extracts induce MM cell death, implicates ferroptosis in MM cell demise. This further highlights plant-derived extracts as promising tools for triggering ferroptosis in MM.

Shikonin (SHK), a natural compound isolated from Lithospermum erythrorhizon roots, exhibits diverse bioactivities, including antioxidant, anti-inflammatory, and antitumor effects, and functions as a proteasome inhibitor and necroptosis inducer (Guo et al., 2019; Guo et al., 2023 Y.; Yan et al., 2023). Shikonin exerts anti-angiogenic effects by downregulating the PI3K/AKT and MAPK signaling pathways, and also displays anticancer activity by targeting ROS (Anti-angiogenic effect of Shikonin, 2020). Studies demonstrate that SHK treatment promotes lactate dehydrogenase release, triggers cell death, induces oxidative stress, and elevates ferrous iron and lipid peroxidation levels. Notably, ferroptosis inhibitors reverse SHK-induced cytotoxicity, confirming ferroptosis involvement. Further mechanistic studies revealed that glutamic-oxaloacetic transaminase 1 (GOT1) serves as a key mediator of SHK-driven ferroptosis by promoting ferritinophagy. These findings underscore SHK’s potential as a therapeutic agent for MM (Li W. et al., 2022).

Apigenin (Api), an edible plant-derived flavonoid, has been recognized as an antitumor agent in numerous experimental and biological studies. It demonstrates cytostatic and pro-apoptotic effects across various malignancies through modulation of multiple signaling pathways (Imran et al., 2020; Jiang Z.-B. et al., 2021; Shi et al., 2023; Sudhakaran et al., 2023; Naponelli et al., 2024). A recent study demonstrated that Api significantly inhibits the progression of bladder cancer by targeting VEGF-β (Apigenin inhibits recurrent bladder cancer, 2025). In addition, it has shown potential therapeutic effects against viral infections (Lee et al., 2023). Notably, our investigation reveals that Api induces myeloma cell apoptosis via the STAT1/COX-2/iNOS signaling axis, demonstrating marked growth inhibition in MM cells with the most pronounced concentration-dependent effect observed in NCI-H929 cells. Importantly, pretreatment with ferrostatin-1 and deferoxamine attenuated Api’s efficacy on NCI-H929 cells by over threefold, strongly suggesting the potential involvement of ferroptosis-related pathways in Api-mediated MM cell death. Further mechanistic investigations are warranted to fully elucidate this regulatory network (Adham et al., 2021a).

Artesunate (ART), a water-soluble hemisuccinate derivative of dihydroartemisinin (DHA), originates from artemisinin–an active compound isolated from Artemisia annua with extensive traditional Chinese medicinal applications and current use as a frontline antimalarial agent (Qian et al., 2021; Strik et al., 2024). ART exhibits both anticancer activity and cytotoxicity against solid tumors and leukemias, having been applied as an adjuvant therapy for diverse malignancies (Vakhrusheva et al., 2022; Hill et al., 2023; Fan et al., 2024). ART exerts its antitumor effects through multiple pathways, including PPARγ-SCD and PI3K/AKT/FKHR signaling (Xu et al., 2022; FABP5, 2024). Liang et al. investigated ART’s effects on cellular growth, ROS generation, Fe^2+^ levels, lipid peroxidation, and ferroptosis-related gene expression, while further exploring ferroptosis mechanisms in MM cells and their association with sterol regulatory element-binding protein 2 (SREBP2) nuclear localization. ART treatment upregulated ROS, Fe^2+^, and lipid peroxidation levels while suppressing MM cell growth, concomitant with increased ACSL4 expression and decreased GPX4 levels. Notably, ART-induced cell death was reversed by ferroptosis inhibitors ferrostatin-1 (Fer-1) and deferoxamine (DFO). Mechanistically, ART suppressed nuclear localization of SREBP2 in MM cells, accompanied by downregulation of IPP and GPX4, suggesting its capacity to trigger ferroptosis through SREBP2 nuclear localization inhibition and subsequent IPP/GPX4 suppression (Liang et al., 2023).

Eclipta prostrata is a herbaceous plant, and its aerial parts are used as traditional medicine. In traditional Chinese medicine, Eclipta prostrata is employed to treat bleeding, liver diseases, kidney injuries, and snake bites (Fang et al., 2015; Tian et al., 2023). Modern pharmacological studies have shown that Eclipta prostrata exhibits various biological activities, such as anti - tumor, anti - snake venom, anti - inflammatory, antioxidant, and hypolipidemic effects (Timalsina and Devkota, 2021; Yang J. et al., 2023). Its extracts have been found to promote osteogenic differentiation of bone marrow mesenchymal stem cells by targeting METTL3-mediated m6A RNA methylation (Tian et al., 2023). Li et al. found that the ethanol extract of Eclipta prostrata (EEEP) could inhibit the growth of MM cells and induce cell death both *in vitro* and *in vivo*. EEEP triggers ferroptosis in MM cells by promoting the accumulation of MDA and Fe^2+^, lipid peroxidation, and inhibiting GSH. Mechanistically, EEEP - induced lipid peroxidation and MDA accumulation were blocked by the Nrf2 activator NK - 252. EEEP regulates the kelch-like ECH-associated protein 1 (Keap1)/Nrf2/HO - 1 axis and stimulates ferroptosis in MM cells (Li W. et al., 2024).

Thymus vulgaris L. is an important plant that can be used both medicinally and as food. It has demonstrated various pharmacological effects in both traditional medicine and modern medical research. Its main active components include thymol, carvacrol, flavonoids, and volatile oils (Bolatli et al., 2023; Zhou et al., 2023). Arctium lappa L. belongs to the Asteraceae family and has been widely used in traditional Chinese medicine (TCM). It is rich in highly - recognized bioactive metabolites with antioxidant, anticancer, and neuroprotective activities (Yang et al., 2021). Additionally, Arctium lappa L. also shows significant value in nanomedicine applications (Yosri et al., 2023). A study by Adham et al. found that the extracts of Thymus vulgaris (TCF) and Arctium lappa (ACF) can induce cell - cycle arrest and ferroptosis in MM cells and leukemia cells. The researchers observed that myeloma NCI - H929 cells were significantly sensitive to the chloroform fractions of TCF and ACF, and ferroptosis inhibitors eliminated the cytotoxicity of TCF and ACF extracts (Adham et al., 2020).

Andrographolide (AP), a diterpenoid lactone isolated from Andrographis paniculata, is the main component of this plant and exhibits a wide range of pharmacological effects. Multiple studies have demonstrated its potent antitumor activity (Li et al., 2020b; Li et al., 2024 J.; Jiaqi et al., 2023). Andrographolide promotes ferroptosis by inducing mitochondrial dysfunction, thereby inhibiting the development and progression of non-small cell lung cancer (Jiaqi et al., 2023). Research has found that AP can induce the death of MM cells, cause G0/G1 cell - cycle arrest, and trigger oxidative stress responses. These phenomena are accompanied by an increase in the levels of Fe^2+^ and lipid peroxidation in both the cytoplasm and mitochondria. Further research shows that AP may block the Nrf2/HO - 1 signaling pathway by activating P38, thereby inducing ferroptosis. Additionally, the use of ferroptosis inhibitors can rescue the MM cell death induced by AP, indicating a close relationship between AP - induced MM cell death and the ferroptosis pathway (Li W. et al., 2023).

Ginsenoside Rh4 is an active compound extracted from ginseng. It belongs to protopanaxatriol - type ginsenoside saponins and exhibits a variety of pharmacological activities (Wang et al., 2024). It has been proven to improve antibiotic - induced gut microbiota dysbiosis and intestinal inflammation and inhibit the growth of various tumors (Wu et al., 2018; Dong et al., 2023; Jiang et al., 2023; Bai et al., 2024). Ginsenoside Rh4 suppresses the progression of colorectal cancer by modulating gut microbiota-mediated bile acid metabolism. It also inhibits tumor metastasis by targeting signaling pathways such as Wnt/β-catenin and TGF-β/Smad2/3 (Chen et al., 2022a; Bai et al., 2024). A study by Ying et al. found that ginsenoside Rh4 can inhibit the proliferation of MM cells, induce cell apoptosis, promote cell - cycle arrest, etc., and play a key role in inducing ferroptosis in MM cells (Ying et al., 2023). This process may be related to the inhibition of Sirtuin 2 (SIRT2) expression in MM cells by ginsenoside Rh4. SIRT2 regulates the activity of lipid - peroxidation - related enzymes to reduce the occurrence of ferroptosis and may also participate in regulating the ferroptosis sensitivity of myeloma cells by affecting lipid metabolism and antioxidant regulation (Feng et al., 2024).

Fumaria officinalis is a traditional herbal medicine containing various active components and exhibits multiple pharmacological effects such as antioxidant, antibacterial, anti - inflammatory and antispasmodic, as well as immunomodulatory activities (Raafat and El-Zahaby, 2020; Shah et al., 2024). The chloroform and ethyl acetate (EF) fractions of the Fumaria officinalis extract show significant cytotoxic effects on human acute lymphoblastic leukemia cells and MM cells. Among them, EF induces autophagic cell death, while chloroform can stimulate iron - dependent cell death (Adham et al., 2021b).

Nitidine chloride (NC) is an alkaloid extracted from the dried roots of Zanthoxylum nitidum, a plant of the Rutaceae family. It exhibits various pharmacological activities, including antitumor, anti-inflammatory, antimalarial, and antioxidant effects (Lu et al., 2022). NC has been reported to induce caspase-3/GSDME-dependent pyroptosis in lung cancer by inhibiting the PI3K/Akt pathway (Yu et al., 2022). In multiple myeloma (MM), ATP-binding cassette sub-family B member 6 (ABCB6) has been identified as a key protein mediating ferroptosis resistance by maintaining glutathione (GSH) homeostasis and activating the PI3K/AKT signaling pathway. NC targets ABCB6, suppresses the PI3K/AKT pathway, and induces ferroptosis in MM cells (Yin et al., 2023).

Huachansu Injection is derived from the dried skin of the *Bufo gargarizans* and has traditionally been used for its detoxifying, anti-inflammatory, and analgesic properties. It is commonly applied in the treatment of advanced-stage cancers and chronic hepatitis B. Huachansu has been shown to inhibit liver cancer cell growth by reducing the activity and expression of glucose-6-phosphate dehydrogenase (G6PD), thereby disrupting the pentose phosphate pathway (Wu Q. et al., 2024). It can also induce apoptosis in gastric cancer cells by increasing ROS levels and suppressing proteasome activity (Deng et al., 2024). Yang et al. were the first to demonstrate that Huachansu Injection induces ferroptosis in multiple myeloma (MM) via the NRF2/HO-1 signaling pathway. Specifically, Huachansu promotes nuclear translocation of NRF2, which activates the transcription of antioxidant genes such as HO-1. HO-1 catalyzes the degradation of heme, leading to the release of free iron, which triggers the Fenton reaction and the accumulation of lipid peroxides such as MDA, ultimately initiating ferroptosis. Moreover, Huachansu downregulates the expression of SLC7A11 and GPX4, weakening the cellular capacity to eliminate lipid peroxides and thereby preventing the suppression of ferroptosis (Yang et al., 2025) (Table 2).

**TABLE 2:** Summary of the mechanisms by which natural products intervene in MM through regulating ferroptosis.

Active compounds of TCM	Chemical structure	Molecular formula	CAS number	Animal or cell type	Dosage of drugs used	anti-tumor mechanism	References
Shikonin	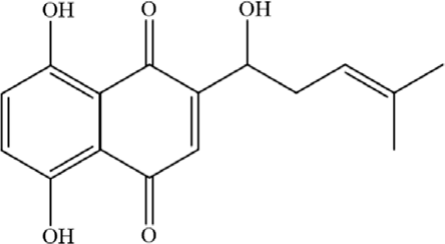	C_16_H_16_O_5_	517–89–5	The human MM cell lines RPMI 8226 and U266; Nod-SCID mice of SPF grade	4 mg/kg	Ferroptosis and immunogenic cell death are induced in MM cells via GOT1-mediated ferritinophagy	Li et al. (2022b)
Apigenin	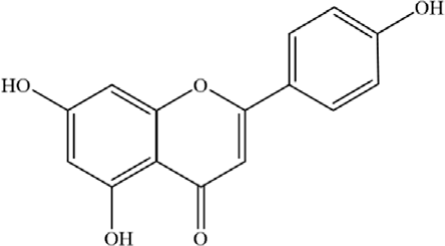	C_15_H_10_O_5_	520–36–5	MM cell line NCI-H929	5, 10, 20 or 40 μM	Apigenin can induce ferroptosis in the MM NCI-H929 cell line, but the specific mechanism requires further investigation	Adham et al. (2021a)
Artesunate	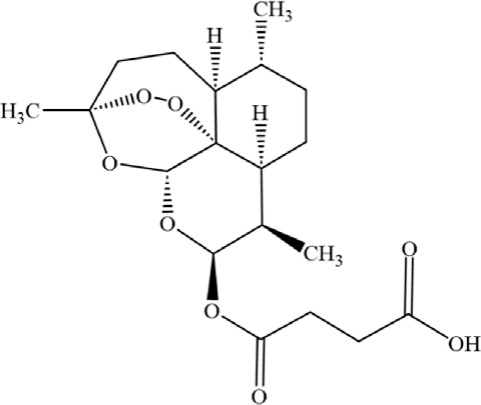	C_19_H_28_O_8_	88495–63–0	The human MM cell lines MM1S (Cat. No. iCell-h291), RPMI8226 (Cat. No. iCell-h183)	40 μM	Artesunate can trigger ferroptosis in MM by inhibiting the nuclear localization of SREBP2 and downregulating IPP and GPX4	Liang et al. (2023)
Ethanol extract of Eclipta prostrata	--	--	--	The human MM cell lines RPMI-8226 and U266, Nod-SCID mice of SPF grade	50 mg/kg、100 mg/kg	Eclipta prostrata can induce ferroptosis in MM cells through the Keap1/Nrf2/HO-1 axis	Li et al. (2024c)
Thymus serpyllum extract	--	--	--	MM cell line NCI-H929	0.001–100 μg/mL	The ferroptosis inhibitors abrogated cytotoxicity of the extracts, indicating that ferroptosis played a role in the process of cell death	Adham et al. (2020)
Arctium lappa extract	--	--	--	MM cell line NCI-H929			
Andrographolide	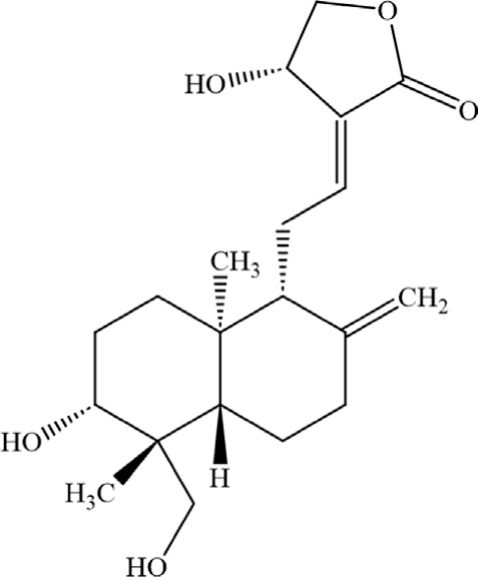	C_20_H_30_O_5_	5508–58–7	The human MM cell lines RPMI-8226 and U266	20 or 40 μM	Andrographolide induced ferroptosis in multiple myeloma cells by regulating the P38/Nrf2/HO-1 pathway	Li et al. (2023b)
Ginsenoside Rh4	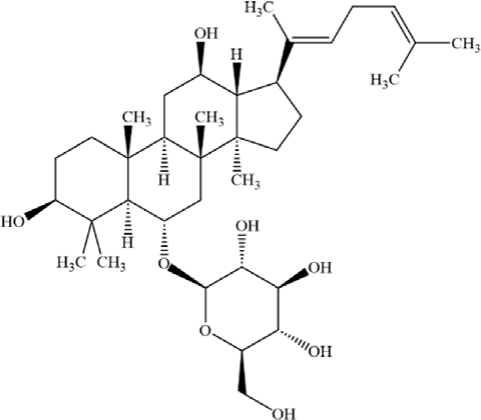	C_36_H_60_O_8_	174721–08–5	MM cell line NCI-H929	25, 50, 100 and 200 μM	Ginsenoside Rh4 induces ferroptosis in MM and inhibits its malignant progression by regulating SIRT2	Ying et al. (2023)
Nitidine chloride	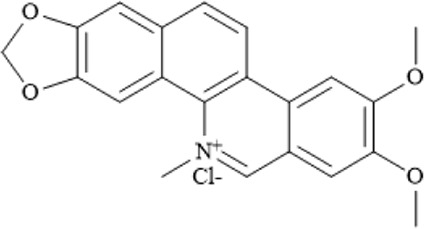	C_21_H_18_ClNO_4_	13063–04–2	The human MM cell lines RPMI-8226 and U266, mouse MM SP2/0 cells and mouse models	2, 4, 6 and 8 μM; 4.5 mg/kg, 6 mg/kg	Nitidine chloride induces ferroptosis in MM cells by targeting ABCB6 and suppressing the PI3K/AKT pathway	Yin et al. (2023)
Huachansu	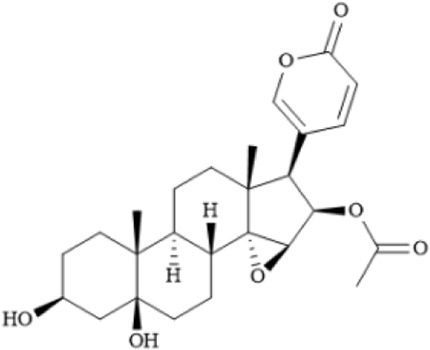	C_26_H_34_O_7_	1108–68–5	The human MM cell lines ARP-1, CAG and JJN3	0.09 μg/mL, 0.18 μg/mL, 0.36 μg/mL	Huachansu Injection induces ferroptosis in multiple myeloma through NRF2/HO-1 signaling pathway	Yang et al. (2025)

GOT1, Glutamic-oxaloacetic transaminase 1; SREBP2, Sterol regulatory element binding protein 2; IPP, Isopentenyl pyrophosphate; Keap1, kelch-like ECH-associated protein 1; Nrf2, Nuclear factor erythroid 2-related factor 2; HO-1, Heme oxygenase-1; SIRT2, Sirtuin 2; ABCB6, ATP-binding cassette sub-family B member 6.

### 7.2 Proteasome inhibitors

In MM cells, high proteasome activity is a crucial factor for degrading misfolded immunoglobulins to ensure cell survival. As a proteasome inhibitor, bortezomib has been clinically used to treat patients with MM. Bortezomib exerts its therapeutic effects in MM by binding to the β5 subunit of the 26S proteasome to inhibit the ubiquitin–proteasome pathway, blocking activation of the Nuclear factor kappa-light-chain-enhancer (NF-κB) of activated B cells signaling pathway, inducing mitochondrial apoptosis, and suppressing angiogenesis and bone destruction within the tumor microenvironment. However, the autophagic process activated by the accumulation of intracellular misfolded immunoglobulins leads to bortezomib resistance in multiple myeloma (Cengiz Seval and Beksac, 2018).

MM cells exhibit higher intracellular iron and ferritin levels compared to normal cells. Bortezomib can trigger ferritin degradation, increase intracellular free Fe^2+^, and promote ferroptosis in MM cells. Further mechanistic studies have shown that bortezomib effectively increases the level of NCOA4 by preventing proteasomal degradation, confirming its role in enhancing ferritinophagy. Moreover, the combined use of bortezomib and the ferroptosis inducer RSL - 3 can synergistically promote ferroptosis in MM cell lines. The synergistic effect of bortezomib and RSL - 3 in MM cells can be successfully counteracted by using the ferroptosis inhibitor liproxstatin - 1 (Zhang et al., 2024f).

Treatment with docosahexaenoic acid or eicosapentaenoic acid (DHA/EPA) can enhance the sensitivity of MM cells to bortezomib. Compared with the combined treatment of DHA/EPA and bortezomib, the sole application of DHA/EPA can significantly reduce the intracellular GSH level and alter the expression of metabolites and key enzymes related to GSH metabolism. RNA - seq indicates that DHA/EPA may enhance the efficacy of bortezomib by activating ferroptosis - related signaling pathways, suggesting that ferroptosis may play a positive role in improving drug sensitivity and overcoming drug resistance in the treatment of MM (Chen J. et al., 2021).

In conclusion, ferroptosis - related genes may become important factors for MM prognosis assessment, and more relevant genes still need to be explored through experiments. Although the above evidence proves the important role of ferroptosis in MM, further research is still needed to determine the ferroptosis inducers applicable in the human body, as well as their concentrations and application methods, in order to exploit the potential of ferroptosis as a new and generalizable clinical strategy.

### 7.3 Immunomodulatory drugs

Fingolimod (FTY720) was initially used as an immunosuppressant for the treatment of multiple sclerosis (Cengiz Seval and Beksac, 2018). Fingolimod exerts its therapeutic effects in multiple sclerosis by activating sphingosine-1-phosphate receptors (S1PRs) and inducing their internalization, thereby blocking lymphocyte egress from lymphoid tissues to the periphery. This reduces the infiltration of autoreactive T cells into the central nervous system, ultimately attenuating inflammatory damage and slowing disease progression (Kappos et al., 2010). However, subsequent studies have revealed its extensive anti - tumor effects (Hasan Ali et al., 2020). Research has shown that FTY720 can reduce the mRNA and protein levels of GPX4 and SLC7A11 in MM cells. As key regulators of ferroptosis, GPX4 and SLC7A11 are highly expressed in MM cells, indicating that the induction of MM cell death by FTY720 is likely related to the ferroptosis pathway. Further studies have demonstrated that FTY720 activates PP2A to dephosphorylate the AMP - activated protein kinase subunit α (AMPKα) and reduces the expression of phosphorylated eukaryotic elongation factor 2 (eEF2), ultimately leading to MM cell death. FTY720 induces ferroptosis and autophagy through the PP2A/AMPK signaling pathway, and these two modes of cell death can promote each other, providing a new strategy for the treatment of MM (Zhong et al., 2020).

### 7.4 Other antitumor chemicals

Adapalene (ADA), a third-generation retinoid, is commonly used for the treatment of acne vulgaris. ADA selectively activates retinoic acid receptors (RAR-β/γ), thereby inhibiting excessive proliferation and differentiation of keratinocytes, reducing follicular hyperkeratinization, and downregulating inflammatory cytokines such as IL-6, ultimately contributing to its therapeutic effects in acne (Waugh et al., 2004). Recent studies have shown that ADA induces dose-dependent cell death in MM cells (Cao et al., 2024). ADA induces ferroptosis in MM cells by downregulating the protein expression of GPX4 and SLC7A11, which are key ferroptosis marker proteins. Moreover, studies have found that ADA is beneficial in restoring the sensitivity of cells from patients with bortezomib - resistant relapsed or refractory MM to bortezomib. ADA can disrupt the activation of the NF-κB pathway triggered by bortezomib and promote cell death in the bortezomib - resistant MM subpopulation (Cao et al., 2024).

Zhang et al. investigated the effects of the novel methyltransferase G9a inhibitor DCG066 on MM cells. They found that after DCG066 intervention, the levels of ROS, iron, and MDA in MM cells increased significantly, while the level of GSH decreased. The protein expression levels of SLC7A11, GPX4, Nrf2, and HO - 1 were significantly reduced. These phenomena could be reversed by the ferroptosis inhibitor Ferrostatin-1 and the Nrf2 activator tert - butylhydroquinone, which confirmed that DCG066 can inhibit the proliferation of MM cells and induce ferroptosis through the Nrf2/HO - 1 signaling pathway (Zhang et al., 2024g).

Disulfiram (DSF) is an FDA-approved drug for the treatment of alcohol dependence. It exerts its effect by inhibiting aldehyde dehydrogenase (ALDH), thereby blocking the conversion of ethanol to acetic acid. This leads to the accumulation of acetaldehyde in the body, causing adverse reactions such as nausea, vomiting, and palpitations, which help establish an aversive response to alcohol consumption (Heather, 1989). In recent years, DSF has shown emerging therapeutic potential in hematological malignancies due to its low toxicity and multi-target properties (Weiser Drozdkova and Smesny Trtkova, 2021). Arkan et al. found that treatment with disulfiram (DSF) increased the production of cytoplasmic and mitochondrial ROS in MM cells, led to the loss of mitochondrial membrane potential and an increase in the level of lipid peroxidation, and caused a significant downregulation of ferroptosis - related genes including GPX4 (Arkan and Akcora-Yildiz, 2025). The ferroptosis inhibitor liproxstatin - 1 could alleviate DSF - induced ferroptosis in MM cells by promoting the upregulation of GPX4. DSF can also overcome carfilzomib resistance by increasing the level of lipid peroxidation and exert a synergistic effect with carfilzomib in carfilzomib - resistant MM cell lines, indicating the potential of DSF in the treatment of MM.

T-5224 is an activator protein-1 (AP-1) inhibitor that specifically blocks the DNA-binding activity of the c-Fos/c-Jun heterodimer, thereby suppressing the expression of downstream pro-inflammatory cytokines such as TNF-α and matrix metalloproteinases like MMP-3. Through this mechanism, T-5224 exerts anti-inflammatory, antioxidant, and anti-degenerative effects (Sasakura et al., 2024). Previous studies have shown that T - 5224 can inhibit the proliferation of MM cells and induce their apoptosis (Tang et al., 2023). Tang et al. found that T - 5224 - induced MM cell death could be reversed by the ferroptosis - specific inhibitor ferropstatin-1, and T - 5224 reduced the protein levels of GPX4 and SLC7A11, the key regulators of ferroptosis in MM cells. Further research revealed that T - 5224 reduced the phosphorylation of components in the PI3K and AKT signaling pathways, suggesting that T - 5224 may induce ferroptosis in MM cells by regulating the PI3K/AKT signaling pathway (Tang S. et al., 2024) (Table 3).

**TABLE 3:** Mechanisms of antitumor chemicals in treating MM through regulation of ferroptosis-related pathways.

Anti-tumor chemical	Chemical structure	Molecular formula	CAS number	Animal or cell type	Dosage of drugs used	Anti-tumor mechanism	References
Bortezomib	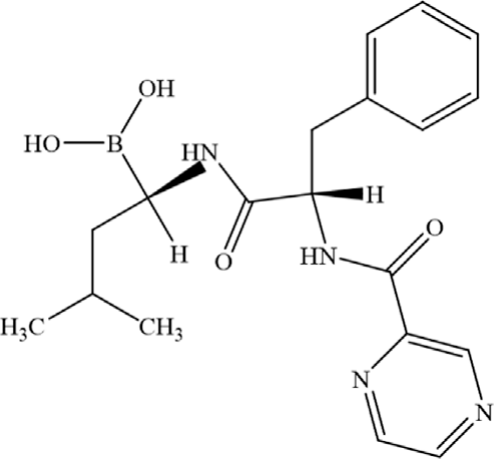	C_19_H_25_BN_4_O_4_	179324–69–7	Human MM cell lines ARP1 and OCI-My5	1.5 nM、3 nM	Bortezomib may enhance NCOA4-mediated ferritinophagy by inhibiting the proteasome, thereby increasing free Fe^2+^ levels and promoting ferroptosis in MM cells	Zhang et al. (2024f)
Fingolimod (FTY720)	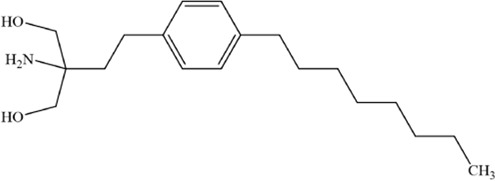	C_19_H_33_NO_2_	162359–55–9	Human MM cell lines U266 and RPMI8226	5、10、15 μM	FTY720 induces ferroptosis and autophagy via PP2A/AMPK pathway in MM cells	Zhong et al. (2020)
Adapalene	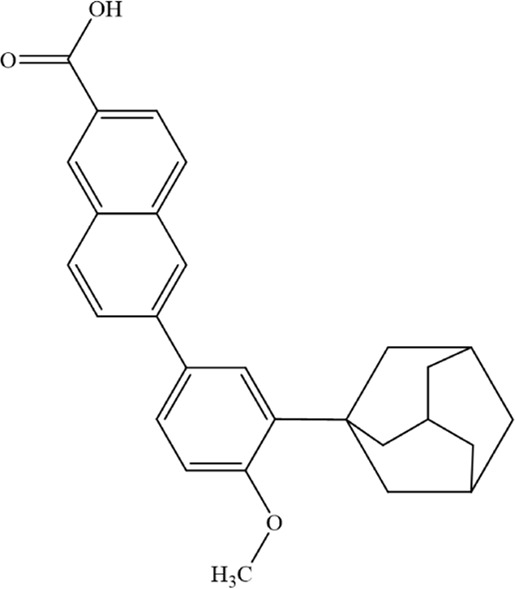	C_28_H_28_O_3_	106685–40–9	Human H929 and LP-1 MM cell lines	20、40、60、80、100 μmol/L	Adapalene induces ferroptosis in MM cells by downregulating the protein expression of GPX4 and SLC7A11	Cao et al. (2024)
DCG066	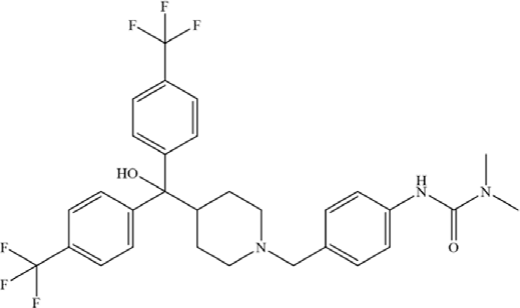	C_30_H_31_F_6_N_3_O_2_	494786–13–9	Human MM cell lines ARH-77 and RPMI-8226	5 µM	DCG066 inhibits MM proliferation and induces ferroptosis via the Nrf2/HO-1 pathway	Zhang et al. (2024g)
Disulfiram	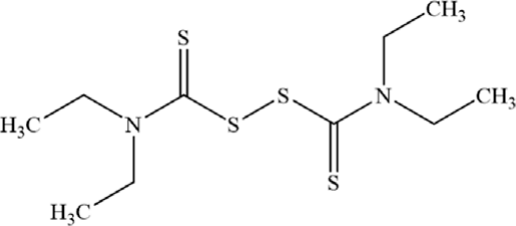	C_10_H_20_N_2_S_4_	97–77–8	Human MM cell lines NCI H929, U266, and RPMI 8226	5、10、25、50 µM	DSF induces ferroptosis in MM cells by downregulating GPX4, increasing cytosolic and mitochondrial ROS, and elevating lipid peroxidation	Arkan and Akcora-Yildiz (2025)
T-5224	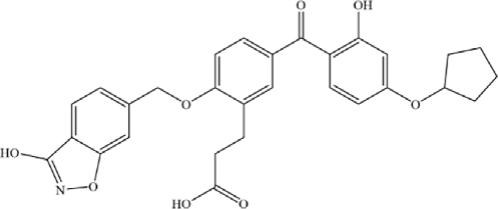	C_29_H_27_NO_8_	530141–72–1	Human MM cell lines ARP1 and RPMI 8226; Nod-SCID mice of SPF grade	10、20 µM (*in vitro*) 20 mg/kg (*in vivo*)	T-5224 reduces the protein levels of key ferroptosis regulators GPX4 and SLC7A11 in MM cells and induces ferroptosis in MM cells through the PI3K/AKT pathway	Tang et al. (2024a)

NCOA4, Nuclear receptor coactivator 4; PP2A, Protein phosphatase 2A; AMPK, AMP activated protein kinase; DSF, Disulfiram.

In another study, MM cells harboring the t (4; 14) translocation exhibited marked sensitivity to Class II ferroptosis inducers (Class II FINs), whereas t (4; 14)-negative cells were resistant. This selective cytotoxicity is attributed to the overexpression of MMSET, a histone methyltransferase upregulated due to the t (4; 14) translocation. MMSET enhances transcriptional activation of ACSL4, facilitating the incorporation of polyunsaturated fatty acids (PUFAs) into membrane phospholipids. PUFA enrichment increases oxidative damage susceptibility, thereby heightening ferroptosis sensitivity in t (4; 14)-positive MM cells.*In vitro* assays confirmed that combining Class II FINs with bortezomib results in enhanced ferroptosis induction and significantly reduced viability of t (4; 14)-positive MM cells. As a genetically guided therapeutic strategy, this approach holds great clinical promise. Moreover, MMSET and ACSL4 expression levels may serve as predictive biomarkers for Class II FIN responsiveness, providing a basis for patient stratification (Zhang J. et al., 2024).

## 8 Regulatory effects of existing MM therapies on the ferroptosis pathway

Currently, no drugs directly targeting the ferroptosis pathway have been approved for the treatment of MM. This review summarizes investigational agents targeting ferroptosis in MM and highlights recent progress in understanding how existing MM therapies modulate ferroptosis (Table 4).

**TABLE 4:** Development of MM therapeutic agents targeting ferroptosis-related pathways and summary of studies on ferroptosis regulation by approved MM drugs in other diseases.

Drug Name	Drug Type	Development Stage	Mechanism of Action in Ferroptosis	Target Disease	Study Type	Reference
Bortezomib (combined with RSL3)	Proteasome Inhibitors	Approved Drug	Inhibition of proteasome activity, activates NCOA4-mediated ferritinophagy, leading to the release of free Fe^2+^ and enhanced accumulation of lipid peroxides	RRMM	Clinical trial, *in vitro* experiment	Zhang et al. (2024f)
DHA/EPA	Omega-3 Fatty Acids	Drug in preclinical study	Significantly reduces intracellular GSH levels, leading to GPX4 inactivation and accumulation of lipid peroxides, thereby promoting ferroptosis; reverses bortezomib resistance	MM resistant to bortezomib	*In vitro* experiment, *in vivo* experiment	Chen et al. (2021a)
Fingolimod	Immunomodulatory Drugs	Approved Drug	Reduces the expression of GPX4 and SLC7A11 in MM cells and induces ferroptosis and autophagy via the PP2A/AMPK pathway	MM	*In vitro* experiment	Zhong et al. (2020)
Adapalene	Third-generation retinoid	Approved Drug	Adapalene induces ferroptosis by downregulating GPX4 and SLC7A11 expression, leading to glutathione depletion and lipid peroxide accumulation	MM resistant to bortezomib	*In vitro* experiment	Cao et al. (2024)
DCG066	Methyltransferase G9a inhibitor	Drug in preclinical study	DCG066 activates the Nrf2/HO-1 pathway, reduces the levels of GSH, SLC7A11, and GPX4, increases ROS and MDA accumulation, and induces ferroptosis	MM	*In vitro* experiment	Zhang et al. (2024g)
Disulfiram	Aldehyde dehydrogenase inhibitor	FDA-approved; preclinical in MM	Disulfiram induces ferroptosis by inhibiting GPX4 activity and promoting ROS and lipid peroxide accumulation, primarily through disruption of redox homeostasis	MM BCR-ABL + leukemia Myelodysplastic syndromes Nasopharyngeal Cancer Triple-Negative Breast Cancer Hepatocellular carcinoma Non-small cell lung cancer Glioblastoma Bladder Cancer	Clinical trial, *in vitro* experiment, *in vivo* experiment	Li et al. (2020c), 2024a; Qiu et al. (2020), Ren et al. (2021), Chu et al. (2023), Liu et al. (2024), Sharma et al. (2024), Zeng et al. (2024)
T-5224	Activator protein-1 inhibitor	Drug in Clinical Trial	T-5224 reduces the protein levels of GPX4 and SLC7A11 in MM cells and induces ferroptosis through the PI3K/AKT pathway	MM	*In vitro* experiment, *in vivo* experiment	Tang et al. (2024a)
Class II FINs	Ferroptosis inducers	Drug in preclinical study	Class II FINs target the t (4; 14) translocation–driven MMSET–ACSL4 axis to promote the synthesis of PUFA-PLs, thereby enhancing lipid peroxidation sensitivity. In combination with bortezomib, they further induce ferroptosis in t (4; 14)-positive MM cells by increasing Fe^2+^ release and suppressing GPX4-mediated antioxidant defense	t (4; 14)-positive MM	Clinical trial, *in vitro* experiment, *in vivo* experiment	Zhang et al. (2024c)
Carfilzomib	Proteasome Inhibitors	Approved Drug	The combination of carfilzomib and^125^I seed radiation promotes ferroptosis by enhancing intracellular Fe^2+^ accumulation and downregulating GPX4 expression	Esophageal squamous cell carcinoma	*In vitro* experiment, *in vivo* experiment	Wang et al. (2025)
PROTAC-PD-Q2	--	Treatment in preclinical study	The PROTAC-PD-Q2 probe induces ferroptosis by targeting GPX4 for degradation via the ubiquitin–proteasome system, thereby impairing its ability to repair lipid peroxides, leading to ROS accumulation and triggering a Fenton reaction cascade	--	*In vitro* experiment	Zhu et al. (2023)
Selinexor	Selective Inhibitor of Nuclear Export	Approved Drug	In CML, selinexor inhibits the nuclear export protein XPO1 to block the NF-κB signaling pathway, downregulate GPX4 expression, and activate NCOA4-mediated ferritinophagy. In combination with ferroptosis inducers, it enhances lipid peroxidation, thereby overcoming drug resistance and inducing ferroptosis	Chronic myeloid leukemia	*In vitro* experiment	Cui et al. (2024)
Venetoclax	BCL-2 inhibitor	Approved Drug	Venetoclax induces apoptosis by inhibiting BCL-2 and indirectly enhances the sensitivity of AML cells to ferroptosis. When combined with ferroptosis inducers, it synergistically promotes lipid peroxide accumulation, overcoming the antioxidant defense threshold of resistant cells	Acute myeloid leukemia	*In vitro* experiment, *in vivo* experiment	Shi et al. (2025)

RRMM, Relapsed/refractory multiple myeloma; DHA/EPA, Docosahexaenoic acid or eicosapentaenoic acid; MDA, Malondialdehyde; FDA, Food and Drug Administration; MMSET, MM SET domain-containing protein; PROTAC, PROteolysis targeting chimera; CML, Chronic myeloid leukemia; XPO1, Exportin 1; NF-κB, Nuclear factor kappa-light-chain-enhancer of activated B cells; BCL-2, B-cell lymphoma-2; AML, Acute myeloid leukemia.

Studies have shown that the combination of the proteasome inhibitor carfilzomib and iodine-125 (^125^I) seed radiation exhibits potent antitumor effects. Mechanistically, ^125^I seed radiation induces intracellular accumulation of Fe^2+^ and lipid peroxides, but also upregulates ferroptosis suppressors SLC7A11 and GPX4. The combination therapy enhances intracellular Fe^2+^ accumulation and downregulates GPX4 expression, thereby promoting ferroptosis. However, current evidence is limited to solid tumors, and its efficacy in MM remains to be further investigated (Wang et al., 2025). In addition, *in vitro* experiments suggest that ferroptosis inducers RSL3 and ML162 may synergistically enhance the cytotoxicity of bortezomib and lenalidomide in the MM cell line RPMI-8226, underscoring the therapeutic relevance of ferroptosis-associated pathways in MM (Gao et al., 2023). Pomalidomide, the third-generation IMiD following thalidomide and lenalidomide, has recently been employed in a PROteolysis TArgeting Chimera (PROTAC) strategy. A PROTAC probe named PD-Q2, composed of pomalidomide and the GPX4 inhibitor ML162, effectively induces degradation of GPX4 via the ubiquitin–proteasome system, leading to ROS accumulation and significant suppression of MM cell growth. These findings suggest that GPX4-targeting PROTACs hold promise as novel agents for ferroptosis-based therapy in MM (Zhu et al., 2023).

Selinexor is a small-molecule inhibitor that targets the nuclear export protein Exportin 1 (XPO1). Selinexor-based combination regimens have been recommended by multiple clinical guidelines for the treatment of relapsed/refractory multiple myeloma. In addition, selinexor can block nucleocytoplasmic transport signals in leukemia stem cells and when combined with imatinib, induces cell death in chronic myeloid leukemia (CML) cells. However, drug resistance frequently leads to disease progression. Cui et al. identified a subpopulation within selinexor-resistant cells that exhibits cancer stem cell–like properties, characterized by reduced GPX4 activity and decreased SLC7A11 expression, resulting in impaired GSH synthesis. These cells are resistant to apoptosis-inducing agents but highly sensitive to ferroptosis inducers. Further investigation revealed that co-treatment with selinexor and the ferroptosis inducer RSL3 synergistically eliminates resistant cells through dual mechanisms: inhibition of nuclear export and induction of lipid peroxidation. This study, using single-cell analysis, was the first to reveal a connection between selinexor resistance and ferroptosis evasion in CML, suggesting a novel strategy to reverse drug resistance through ferroptosis induction in hematological malignancies such as MM (Cui et al., 2024).

Venetoclax, a BCL-2 inhibitor, promotes apoptosis in tumor cells. Preclinical data have shown its promising efficacy in both newly diagnosed MM and t (11; 14)-positive MM subtypes (Kaufman et al., 2021). Scavenger receptor class B type 1 (SR-B1), a key receptor in cholesterol metabolism, is significantly upregulated in acute myeloid leukemia (AML) and correlates with poor prognosis. SR-B1 deletion reduces cellular cholesterol uptake and inhibits GPX4 activity. Moreover, SR-B1 deficiency disrupts cholesterol homeostasis, enhances PUFA oxidation, increases lipid ROS, and triggers ferroptosis, while also downregulating BCL-2 expression. Further studies revealed that SR-B1 inhibition can reverse venetoclax resistance and promote AML cell death through ferroptosis. Targeting SR-B1 offers a novel therapeutic strategy in hematologic malignancies. The combination of the SR-B1 inhibitor Block Lipid Transport-1 (BLT-1) and venetoclax has entered a Phase I clinical trial (Shi et al., 2025).

Collectively, these findings highlight the emerging significance of ferroptosis as a therapeutic vulnerability in hematologic malignancies. The integration of ferroptosis-targeted strategies—either through small-molecule inducers, pathway modulators, or combination therapies—may offer novel avenues to overcome drug resistance and improve treatment outcomes in MM and related disorders.

## 9 Toxicology and side effects

Current evidence reveals an intrinsic paradox between therapeutic efficacy and safety profiles of bioactive compounds - no identified pharmacologically active agent completely avoids non-specific off-target effects on healthy tissues (Guo C. et al., 2023). While natural products demonstrate multi-component synergism with multi-target and multi-pathway regulatory effects on ferroptosis in MM intervention, their complex phytochemical matrices inherently harbor potential toxicological risks and adverse reaction profiles. SHK, as an inhibitor of uridine 5′-diphosphateglucuronosyltransferase, exerts toxic effects on liver tissues (Cheng et al., 2019). An *in vitro* study demonstrated that shikonin has potent cytotoxicity against 15 types of cancer cells through mitochondrial dysfunction and can induce apoptosis. However, in the same study, shikonin was also found to be toxic to normal cell lines (Wiench et al., 2012). Another study revealed that shikonin induces erythrocyte death by influencing Ca^2+^ influx and ceramide formation (Lupescu et al., 2014). SHK has also been identified as a mixed and competitive inhibitor of cytochrome P450 enzymes, which may lead to an increased risk of drug interactions and toxicity since these enzymes play a crucial role in drug metabolism (Lupescu et al., 2014). In addition, when administered orally, the LD50 value of shikonin is greater than 1 g/kg, indicating relatively low toxicity. In contrast, the LD50 values for intraperitoneal and intravenous injections are 20 mg/kg and 16 mg/kg respectively, suggesting higher toxicity (Yadav et al., 2022). These results indicate that the administration route of shikonin has a significant impact on its toxicity level. The oral route results in lower absorption rate and less toxicity due to first-pass metabolism, while the injection routes are more toxic as the drug directly enters the circulatory system. Other factors such as metabolism, toxicokinetics, and experimental uncertainties can also affect the drug’s toxicity level.

AP demonstrates potential therapeutic efficacy in MM management, though its toxicity profile and adverse effects warrant critical consideration, particularly regarding cellular, reproductive, and renal impacts. Acute toxicity studies identify 500 mg/kg oral AP as the maximum safe dose (Al Batran et al., 2013), while contrasting evidence shows non-toxicity at 5 mg/kg but exacerbated CCl4-induced hepatotoxicity and weight loss at 50 mg/kg in healthy mice (Lin et al., 2018). A meta-analysis documents 26 cases of AP-induced acute kidney injury manifesting as abdominal pain, nausea/vomiting, and oliguria, with histopathology suggesting acute tubular necrosis (Zhang et al., 2014). Reproductive toxicology reveals that prolonged 25–50 mg/kg AP administration in male rats induces sperm count reduction, motility impairment, and teratozoospermia - though this observation suggests potential male contraceptive applications (Akbarsha and Murugaian, 2000). Synergistic pharmacological interactions emerge with Api enhancing antitumor drug efficacy while reducing toxicity: gemcitabine-Api coadministration exerts superior tumor growth inhibition compared to monotherapy in neoplastic cell lines (Johnson and Gonzalez de Mejia, 2013), yet paradoxically, kaempferol-Api combination only suppresses tumor growth *in vitro* while increasing tumor burden *in vivo* (Tang et al., 2017). Adapalene exhibits oral LD50 > 10 mL/kg in rodents, demonstrating retinoid-like side effects during chronic use. Dose-dependent correlations emerge for ovarian gonadomas/thyroid carcinomas in female rats and benign/malignant adrenal medullary pheochromocytomas in males during oral trials (Piérard et al., 2009).

In conclusion, while AP, SHK, ADA, and related compounds exhibit multi-faceted pharmacological potential, their toxicological profiles and adverse effect manifestations remain clinically significant. Elucidating both therapeutic mechanisms and toxicity pathways proves essential for novel therapeutic development and enhancing clinical safety parameters in pharmacological applications.

## 10 Conclusion and outlook

MM, a B-cell-derived malignant clonal proliferation of plasma cells, ranks as the second most prevalent hematologic malignancy, accounting for 15%–20% of hematological cancer-related mortality (Nooka et al., 2024). This clinically heterogeneous entity is characterized by clonal plasma cell expansion and monoclonal immunoglobulin overproduction, with frequent relapse patterns. Ferroptosis, an emerging regulated cell death modality defined by iron-dependent lipid ROS accumulation, has shown growing therapeutic potential in oncology. This review delineates ferroptosis’s multifaceted roles in MM progression through iron metabolism, oxidative stress, and lipid peroxidation pathways, while systematically summarizing experimental evidence of natural product extracts and antineoplastic compounds targeting MM ferroptosis. However, the intricate pathogenesis of MM and its inherent heterogeneity result in complex interplay between ferroptosis signaling pathways, necessitating further mechanistic elucidation of their crosstalk and regulatory networks. Current ferroptosis research in MM remains confined to preclinical investigations, with clinical translation requiring rigorous validation through human trials. Bridging this translational gap between ferroptosis biology and clinical MM therapeutics represents a critical future research frontier.

While NPs demonstrate potential to modulate ferroptosis through multidimensional mechanisms influencing MM progression, critical challenges hinder clinical translation. Firstly, current investigations remain predominantly confined to single-target/pathway mechanistic elucidation, lacking both comprehensive systems pharmacology analyses of network regulation and high-level evidence-based medical validation. The incomplete clinical evidence chain, heavily reliant on *in vitro* studies, necessitates prioritized development of ferroptosis-specific biomarkers and innovation in spatiotemporal controlled-release technologies for ferroptosis inducers. These advancements could accelerate discovery of novel ferroptosis pathway-targeting antitumor compounds. Concurrently, substantial research efforts must elucidate the ferroptosis-modulating mechanisms of existing antineoplastic agents through integrated preclinical-clinical investigations, which is crucial for developing novel effective anti-myeloma therapeutics. Secondly, implementing high-quality multicenter, double-blind randomized controlled trials remains imperative to rigorously evaluate the therapeutic efficacy and safety profile of NPs in modulating ferroptosis-related pathways for MM management, thereby ensuring scientifically robust and clinically translatable outcomes. Thirdly, comprehensive characterization of NPs dose-response relationships is essential to establish optimal therapeutic thresholds while mitigating potential long-term toxicity and safety risks. Enhanced investigation into NP toxicological profiles, coupled with innovations in targeted delivery systems to prolong circulatory residence time, could significantly improve treatment precision. Concurrent standardization of NP quality control protocols must be prioritized to ensure reproducibility across preclinical and clinical studies.

In this review, we summarize investigational agents targeting ferroptosis-related pathways in MM and highlight studies in which approved MM therapies modulate ferroptosis in other diseases. Current evidence suggests that MM cells, particularly those resistant to standard therapies or harboring genetic alterations such as the t (4; 14) translocation, are highly sensitive to ferroptosis. However, clinical translation of this mechanism remains in its infancy.

Future research should focus on developing highly selective and potent ferroptosis inducers suitable for hematologic malignancies. These may include small molecules targeting GPX4, SLC7A11, or ferritinophagy regulators like NCOA4, as well as compounds modulating iron metabolism or lipid peroxidation. PROTAC-based degraders and dual-target agents represent promising next-generation strategies. ACSL4 plays a dual role in MM: it promotes tumor growth by activating lipid synthesis, but also initiates ferroptosis by catalyzing the production of PUFA-CoA, a key substrate for lipid peroxidation (Zhang et al., 2023). Notably, ACSL4-deficient MM cells exhibit resistance to RSL3, while ACSL4-high cells are highly sensitive, supporting the rationale for targeting ferroptosis in ACSL4-overexpressing MM subtypes to overcome chemoresistance. Combination therapies also hold great potential. Ferroptosis inducers may synergize with proteasome inhibitors, immunomodulatory drugs, or BCL-2 inhibitors to enhance cytotoxicity and overcome resistance. Molecular stratification based on ferroptosis regulators such as MMSET or ACSL4 could further guide personalized treatment approaches. Finally, emerging technologies—including single-cell sequencing, spatial transcriptomics, and CRISPR-based functional screening—are expected to reveal the ferroptosis regulatory network within the MM tumor microenvironment. Advances in nanomedicine and targeted delivery systems will also be essential to improve therapeutic specificity and reduce toxicity.

However, several critical gaps and challenges remain in current research. The dynamic regulatory mechanisms of the tumor microenvironment (TME) are still unclear. The MM bone marrow microenvironment comprises diverse cell types and soluble factors. Recent studies have shown that GDF-15 secreted by M2-polarized tumor-associated macrophages promotes ferroptosis resistance in AML cells by modulating the SLC7A11/GPX4 axis (Lu and Liao, 2025); however, this mechanism has not yet been validated in MM, and the roles of other immune cells within the TME remain unexplored. Moreover, how intercellular communication in the TME dynamically regulates the stability of key ferroptosis molecules such as SLC7A11 and GPX4 remains poorly understood. Another major challenge lies in the genetic heterogeneity of MM. Current studies have only preliminarily revealed that in t (4; 14) translocation-positive patients, MMSET upregulation enhances ACSL4 transcription and ferroptosis sensitivity (Zhang J. et al., 2024). In contrast, ferroptosis regulatory mechanisms in other high-risk subtypes—such as del (17p), t (14; 16), and 1q21 amplification—remain largely unknown. In particular, how epigenetic modifications, such as DNA methylation, influence the expression of ferroptosis-related genes has yet to be elucidated.

Most current studies remain at the *in vitro* stage, which presents inherent limitations. The efficacy, safety, and pharmacokinetic profiles of ferroptosis inducers have not been systematically validated in humans. *In vitro* models fail to recapitulate the complexity of the bone marrow microenvironment and overlook key pharmacokinetic factors such as renal excretion, potentially leading to inaccurate dose prediction. Additionally, drug-resistant cell lines generated *in vitro* do not fully mimic the clonal evolution of tumors during relapse, which may compromise experimental accuracy. In clinical settings, ferroptosis inducers may pose challenges such as hepatotoxicity, immune interference, and off-target cell damage, all of which require optimization through structural refinement and targeted delivery strategies. Moreover, the high heterogeneity of MM remains a major obstacle, and currently, there is a lack of validated biomarkers to predict ferroptosis sensitivity, limiting precise therapeutic stratification.

In summary, targeting ferroptosis offers a novel pharmacological approach for MM treatment. Future research should focus on drug development, combination strategies, mechanistic studies, and biomarker discovery. The synergistic application of natural products (NPs) with chemotherapeutic agents and other treatment modalities may contribute to a more integrated therapeutic framework. Developing safer and more effective ferroptosis-based strategies holds great promise for patients with relapsed or refractory MM. However, the standardization and clinical translation of ferroptosis inducers remain challenging, highlighting the need for continued multidisciplinary efforts to advance precision medicine in MM.

## Glossary

AA: Arachidonic acid
ACF: Arctium lappa
ACSL4: Acyl-CoA synthetase long-chain family member 4
ADA: Adapalene
AdA: Adrenic acid
AIFM2: Apoptosis - inducing factor mitochondria - associated 2
AMPK: AMP-activated protein kinase
Api: Apigenin
ART: Artesunate
BMSCs: Bone marrow mesenchymal stromal cells
BTZ: Bortezomib
CoQ10: Coenzyme Q10
Cys: cysteine
CP: Ceruloplasmin
DHA: Dihydroartemisinin
DHA/EPA: Docosahexaenoic acid or eicosapentaenoic acid
DSF: Disulfiram
EBP: emopamil binding protein
EEEP: ethanol extract of Eclipta prostrata
eEF2: eukaryotic elongation factor 2
eEF2k: eukaryotic elongation factor 2 kinase
EF: Ethyl acetate
FPN1: Ferroportin 1
FRGs: Ferroptosis-related genes
FSP1: Ferroptosis suppressor protein 1
FTY720: Fingolimod
GCL: Glutamate-cysteine ligase
Gln: Glutamine
GLS: Glutaminase
Glu: Glutamate
GLUD1: Glutamate dehydrogenase
GOT1: Glutamic-oxaloacetic transaminase 1
GPX4: Glutathione peroxidase 4
GSH: Glutathione
GSSG: Oxidative glutathione
HCC: Hepatocellular carcinoma
HO-1: heme oxygenase-1
IMiDs: immunomodulatory drugs
IPP: Isopentenyl pyrophosphate
IRF1: Interferon regulatory factor 1
LILRB1: Leukocyte immunoglobulin-like receptor B1
LIP: Labile iron pool
LOXs: Lipoxygenases
LPCAT3: Lysophosphatidylcholine acyltransferase 3
LPOs: lipid peroxides
MDA: Malondialdehyde
MM: Multiple myeloma
MUFAs: Monounsaturated fatty acids
MVA: Mevalonate
NADPH: Nicotinamide adenine dinucleotide phosphate
NPs: Natural products
Nrf2: Nuclear factor erythroid-2 related factor 2
PE: Phosphatidylethanolamine
PIs: Proteasome inhibitors
PLOH: fatty alcohols
PLOOH: Phospholipid hydroperoxides
PP2A: Protein phosphatase 2A
PUFA: Polyunsaturated fatty acid
ROS: Reactive oxygen species
RPE: Retinal pigment epithelial
RT: Radiotherapy
Sec: Selenocysteine
SHK: Shikonin
SLC1A5: Solute carrier family 1 member 5
SLC7A11: Solute carrier family 7 member 11
TCA cycle: Tricarboxylic acid cycle
TCF: Thymus vulgaris
Tf: Transferrin
TfR1: Transferrin receptor 1
TRCs: Tumor-reprogrammed cells
ULK1: UNC-51 like kinase 1
YAP1: Yes-associated protein 1.
